# The Evolutionary Dynamics of Genetic Incompatibilities Introduced by Duplicated Genes in *Arabidopsis thaliana*

**DOI:** 10.1093/molbev/msaa306

**Published:** 2020-11-28

**Authors:** Wen-Biao Jiao, Vipul Patel, Jonas Klasen, Fang Liu, Petra Pecinkova, Marina Ferrand, Isabelle Gy, Christine Camilleri, Sigi Effgen, Maarten Koornneef, Ales Pecinka, Olivier Loudet, Korbinian Schneeberger

**Affiliations:** 1 Department of Chromosome Biology, Max Planck Institute for Plant Breeding Research, Cologne, Germany; 2 Department of Plant Developmental Biology, Max Planck Institute for Plant Breeding Research, Cologne, Germany; 3 Department of Breeding Research, Leibniz Institute of Plant Genetics and Crop Plant Research (IPK) Gatersleben, Stadt Seeland, Germany; 4 Department of Plant Breeding and Genetics, Max Planck Institute for Plant Breeding Research, Cologne, Germany; 5 Department of Cell Biology and Genetics, Faculty of Science, Palacký University Olomouc, Olomouc, Czech Republic; 6 Institut Jean-Pierre Bourgin, INRAE, AgroParisTech, Université Paris-Saclay, Versailles, France; 7 Laboratory of Genetics, Wageningen University & Research, Wageningen, The Netherlands; 8 Institute of Experimental Botany (IEB), Czech Academy of Sciences, Centre of the Region Haná for Biotechnological and Agricultural Research (CRH), Olomouc, Czech Republic; 9 Faculty of Biology, LMU Munich, Planegg-Martinsried, Germany

**Keywords:** genetic incompatibility, duplicated gene, HPA, TIM22, genome-wide association study, loss of function

## Abstract

Although gene duplications provide genetic backup and allow genomic changes under relaxed selection, they may potentially limit gene flow. When different copies of a duplicated gene are pseudofunctionalized in different genotypes, genetic incompatibilities can arise in their hybrid offspring. Although such cases have been reported after manual crosses, it remains unclear whether they occur in nature and how they affect natural populations. Here, we identified four duplicated-gene based incompatibilities including one previously not reported within an artificial Arabidopsis intercross population. Unexpectedly, however, for each of the genetic incompatibilities we also identified the incompatible alleles in natural populations based on the genomes of 1,135 Arabidopsis accessions published by the 1001 Genomes Project. Using the presence of incompatible allele combinations as phenotypes for GWAS, we mapped genomic regions that included additional gene copies which likely rescue the genetic incompatibility. Reconstructing the geographic origins and evolutionary trajectories of the individual alleles suggested that incompatible alleles frequently coexist, even in geographically closed regions, and that their effects can be overcome by additional gene copies collectively shaping the evolutionary dynamics of duplicated genes during population history.

## Introduction

Genetic incompatibilities describe the decrease of fitness due to dysfunctional allele combinations in hybrid individuals (Maheshwari and Barbash 2011). The evolution of genetic incompatibilities has often been explained by the Bateson–Dobzhansky–Muller (BDM) model (Bateson 1909; Dobzhansky 1937; Muller 1942), where independent mutations in interacting genes get fixed in different populations, which cause deleterious epistasis and reduced fitness in their hybrids. Over the past decades, many studies have elucidated the genetic basis of such genetic incompatibilities including reciprocal pseudofunctionalization (i.e., loss of function) of duplicated genes (Fishman and Sweigart 2018; Vaid and Laitinen 2019). Gene duplications can provide genetic backup of essential genes and the basis for evolutionary novelties by allowing for new genetic and epigenetic variations (Conant and Wolfe 2008; Kondrashov 2012; Panchy et al. 2016). However, in some cases, pseudofunctionalization of duplicated essential genes may occur independently in both copies in different individuals. This in turn can lead to the loss of any functional gene copy in the hybrid offspring of such individuals, and thereby cause severe genetic incompatibilities (Lynch and Force 2000).

Genetic incompatibilities introduced by duplicated genes have been reported within inter/intraspecific hybrids of *Arabidopsis thaliana* (Bikard et al. 2009; Durand et al. 2012; Agorio et al. 2017), rice (Mizuta et al. 2010; Yamagata et al. 2010; Nguyen et al. 2017), and *Mimulus* (Zuellig et al. 2018). Identification of these incompatible alleles, however, often relied on genetic mapping in experimental populations, which is a time consuming and costly process. As incompatible alleles at duplicated genes are frequently introduced by loss-of-function (LoF) mutations (such as stop-codon gain, frameshift, gene deletion) and epimutations (Bikard et al. 2009; Blevins et al. 2017), initial examination of LoF (epi)mutations within whole-(epi)genome sequence data could be a shortcut to quickly target promising candidates.

Although several incompatible alleles from duplicated genes have been identified in *A. thaliana*, it is still unclear how these incompatible alleles originate and evolve in natural populations, and how the populations adapt to the reduction in fitness. Untangling the complex evolutionary process would require accurate (epi)genotypes of incompatible genes across sufficiently large natural populations. The Arabidopsis 1001 Genomes Project (Alonso-Blanco et al. 2016) and 1001 Epigenomes Project (Kawakatsu et al. 2016) have released substantial omics data, which can be used to unravel the evolutionary trajectory of such incompatible alleles.

Here, we created an extended version of the Arabidopsis multiparent RIL population (Huang et al. 2011) to identify genetic incompatibilities between several different genotypes simultaneously. Based on distorted segregation of duplicated genes, we mapped four genetic incompatibilities. Unexpectedly, however, we identified several, healthy RILs which carried presumably incompatible allele combinations. Further analysis of their genomes revealed additional gene copies rescuing these severe incompatibilities. Encouraged by this, we searched for incompatible allele combinations within 1,135 accessions of the 1001 Genomes Project (Alonso-Blanco et al. 2016), where these combinations were surprisingly common. Using the incompatible allele combinations as phenotypes, we mapped modifiers of all four incompatibilities using GWA. The LoF alleles from duplicated genes were geographically widely distributed, and coexisted with additional gene copies in the same regions showing how additional gene copies can overcome differential copy loss in a population.

## Results

### Identification of Incompatible Gene Pairs within an Intercross Population

**Fig. 1.**
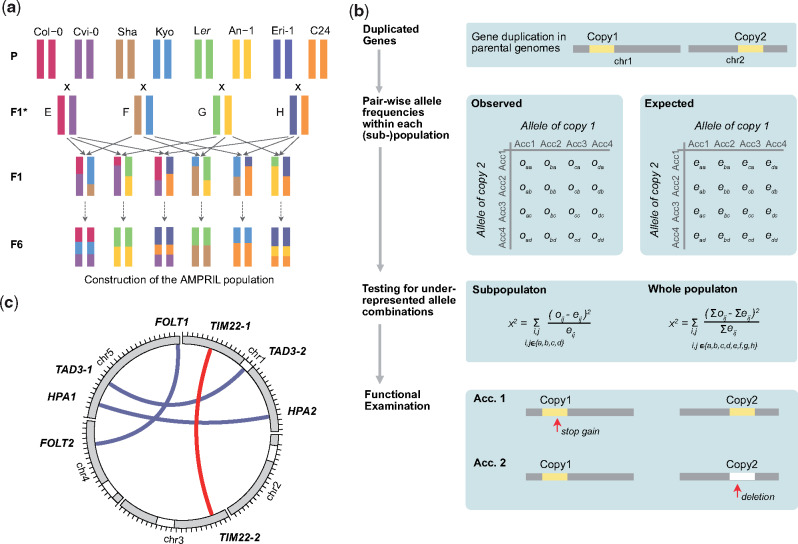
Identification of genetic incompatibilities introduced by duplicated genes in an intercross population. (*a*) Construction of the extended AMPRIL population. Eight different *Arabidopsis thaliana* accessions were used as founder lines. For each of the six subpopulations, two F1* hybrids, which were generated by crossing two founder lines, were again crossed to give rise to the F1 individuals of each population. The F1 individuals were further self-crossed to the F6 generation. (*b*) Workflow for the identification of potentially incompatible alleles in duplicated genes. Unlinked (i.e., on separate chromosomes) duplicated genes were selected and the expected and observed frequencies of all haplotype combinations between the two copies were calculated for each subpopulation and the whole population. Underrepresented allele combinations were identified using χ^2^ test. Each gene duplication with significantly underrepresented allele combinations was evaluated for nonfunctionalized or deleted gene copies in the respective parental genomes. (*c*) The location of incompatible alleles in four duplicated gene pairs identified in the AMPRIL population including one so-far unknown incompatibility (red) and one detected a posteriori in an informed way (*TAD3*).

We used the Arabidopsis Multiparent RIL (AMPRIL) population to find incompatible alleles that arose from duplicated genes. The eight AMPRIL founder accessions (An-1, C24, Col-0, Cvi-0, Eri-1, Kyo, L*er*, and Sha) were selected across the entire geographic distribution of *A. thaliana* including the Northern hemisphere and the Cape Verde Islands. Recently, we generated chromosome-level genome assemblies of all seven, nonreference founder genomes (Jiao and Schneeberger 2020). The first release of the AMPRIL population (AMPRIL I) contained six subpopulations (referred to as ABBA, ACCA, ADDA, BCCB, BDDB, CDDC) derived from reciprocal diallel crosses between four hybrids (A: Col-0 × Kyo, B: Cvi-0 × Sha, C: Eri-1 × An-1, D: L*er* × C24) and the subsequent selfing to the F4 generation by single-seed descent (Huang et al. 2011). Here, we present the extension of the AMPRIL population with six new subpopulations referred to as EFFE, EGGE, EHHE, FGGF, FHHF, and GHHG (E: Col-0 × Cvi-0, F: Sha × Kyo, G: Ler × An-1, H: Eri-1 × C24) based on different diallel intercrossing scheme and selfing of the recombinant genomes until the F6 generation (fig. 1*a*). Each subpopulation consists of approximately 90 individuals representing recombinants of four founders. In total, 992 RILs from all 12 subpopulations were sequenced and analyzed using RAD-seq (Baird et al. 2008) (supplementary data 1, Supplementary Material online) and genotyped with ∼2 million high-quality SNP markers. We used a Hidden Markov Model to reconstruct the parental haplotypes (identity-by-descent) including residual heterozygous regions (Rowan et al. 2015) (supplementary fig. 1 and note 1, Supplementary Material online). The genotyping resulted in 12,878 different recombination breakpoints (on average one breakpoint per 9.3 kb) across the entire population. This allowed us to divide the genome of each progeny into 12,883 haplotype blocks, where each block relates to the haplotype(s) of only one (homozygous regions) or two (heterozygous regions) of the founder haplotypes.

We developed a two-step workflow to combine genetic and genomic evidence to quickly identify incompatible alleles of duplicated genes (fig. 1*b*). In the first step, we selected 781 distal (interchromosome) duplicated gene pairs including 612 gene pairs in which the reference sequence contains two copies and other founder genomes feature at least one copy (supplementary data 2, Supplementary Material online). In the remaining 169 gene pairs, the reference sequence only has one copy and at least one other parental genome has an additional copy in a different chromosome. As genetic incompatibility leads to the underrepresentation of incompatible allele combinations (Ackermann and Beyer 2012; Corbett-Detig et al. 2013), we searched for significant distortions from the expected frequencies of all parental allele combinations across all 781 duplicated gene pairs in all 12 subpopulations, two merged subpopulations (ABBA and EFFE, CDDC and GHHG as they share the same founders), and the whole population (see Materials and Methods). These tests revealed significant distortions in 236 gene pairs (χ^2^ test, *P* value <0.05, multiple testing corrected) in at least one of the populations.

However, the observed distortions do not necessarily result from genetic incompatibilities in the tested gene. Alternatively, such distortions can also occur if the tested gene duplicate is closely linked to a genetic incompatibility. Hence, in a second step, we examined the alleles of the gene pairs in the founder genomes for LoF variations or hypermethylated promoters (fig. 1*b*, see Materials and Methods). This examination revealed three gene pairs with functional disruption in both of the duplicates in at least one of the founder genomes. These three duplicated genes included two, *HISTIDINOL PHOSPHATE AMINOTRANSFERASE* (*HPA*) (Bikard et al. 2009) and *FOLATE TRANSPORTER* (*FOLT*) (Durand et al. 2012), which were already known for their ability to introduce genetic incompatibilities, as well as one gene pair, which so-far was not reported as the genetic basis for a genetic incompatibility, *TIM22* (*TIM22-1*: *AT1G18320*, *TIM22-2*: *AT3G10110*) (fig. 1*c*). For all other 233 gene pairs, we could not identify nonfunctional alleles in both copies.

We noted that another duplicated gene, *tRNA ADENOSINE DEAMINASE 3* (*TAD3*), which is also know to introduce a genetic incompatibility (Agorio et al. 2017), was not considered by our initial testing even though the genotypes of the AMPRIL founders should lead to incompatible allele combinations in the RIL populations: all founder genomes except for Kyo have a functional *TAD3-1* (supplementary table 1, Supplementary Material online), whereas the Kyo *TAD3-1* gene is silenced most likely due to its methylated promoter (similar to the Nok-1 and Est-1 accessions in which the incompatibility was described originally; Agorio et al. 2017). The lack of a functional *TAD3-1* in Kyo is counterbalanced by additional copies (*TAD3-2*) (which however were not part of the main chromosome scaffolds of the Kyo genome assembly). Therefore, the *TAD3* gene duplicate was not considered initially, however, we also used this incompatibility for further analysis.

### Genetic Incompatibility Introduced by Diverged Copies of *TIM22*

**Fig. 2.**
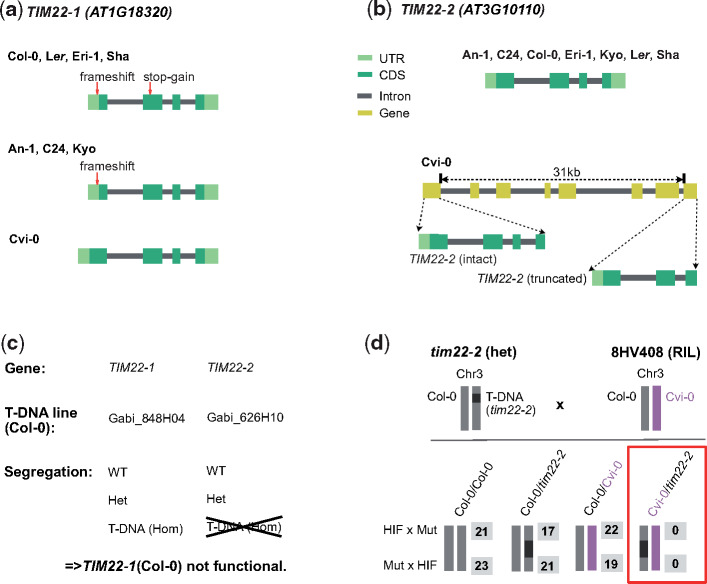
Genomic and genetic evidence of incompatible *TIM22* alleles. (*a*) Gene structure of *TIM22-1* in the genomes of the eight AMPRIL parents. The loss-of-function variants (1-bp frameshift indel and a premature stop-codon) relative to the intact *TIM22-1*^Cvi-0^ are shown. The deleterious effect of the frameshift is erased by an alternative translation start site. (*b*) Gene structure of *TIM22-2* in the genomes of the eight AMPRIL parents. The eight accessions share the structure of *TIM22-2* without recognizable loss-of-function variants, however, a truncated copy of *TIM22* could be found in Cvi-0 ∼31 kb downstream of *TIM22-2*. (*c*) Segregation of T-DNA alleles within the descendance of two segregating Col-0 T-DNA mutant lines (*tim22-1* and *tim22-2*). (*d*) A heterozygous T-DNA line (*tim22-2*) in the Col-0 background (i.e., nonfunctional for *TIM22-1*) was crossed to 8HV408 (heterozygous Col-0/Cvi-0 at *TIM22-2* and homozygous for the Col-0 allele at *TIM22-1*, i.e., nonfunctional for *TIM22-1*). The number of all four possible F1 progenies are shown (in gray) for both cross directions. Although the *TIM22-2^Col-0^* allele can complement the T-DNA, the *TIM22-2^Cvi-0^* could not, implying that the *TIM22-2^Cvi-0^* allele is nonfunctional. HIF, heterogeneous inbred families; Mut, mutant.

Before we started our analysis of the four incompatibilities, we verified that the LoF alleles of *TIM22* are in fact the causal basis of the genetic incompatibility that we observed in the AMPRIL population. *TIM22* encodes for a mitochondrial import inner membrane translocase subunit of the *TIM17*/*TIM22*/*TIM23* family protein (Murcha et al. 2007). All eight founders feature two annotated *TIM22* copies, whereas Cvi-0 includes an extra truncated copy ∼31 kb downstream to *TIM22-2* (fig. 2*a* and *b*). We found significant segregation distortions in the complete AMPRIL population and one subpopulation, EGGE, where the double homozygous allele combination *TIM22-1*^Col-0^*TIM22-2*^Cvi-0^ was significantly underrepresented (supplementary tables 2 and 3, Supplementary Material online).

We observed an in-frame premature stop-codon in Col-0 (mis-annotated in the reference annotation) suggesting that *TIM22-1* is not functional in Col-0 (fig. 2*a* and supplementary table 4, Supplementary Material online). To test if *TIM22-1* is truly nonfunctional in Col-0, we used the segregation of two T-DNA insertion mutants in the two *TIM22* paralogs in Col-0. This showed that *tim22-1* could be homozygous for the T-DNA insertion allele but the T-DNA in *tim22-2* could not be found in homozygous state (fig. 2*c*). This suggests that, in Col-0, *TIM22-1* is not functional and *TIM22-2* is the only functional copy.

The *TIM22* paralogs colocated within the regions of a previously reported genetic incompatibility (hereafter named as LD2: LD2.1 for the locus at chromosome 1 and LD2.3 for the locus at chromosome 3) which was mapped in a Cvi-0 × Col-0 RIL population (Simon et al. 2008). The genetic underpinnings of this incompatibility however were still unknown. This incompatibility was expressed by a striking underrepresentation of homozygous LD2.1^Col-0^ combined with homozygous LD2.3^Cvi-0^, which was in agreement with the reduced allele combinations in the AMPRIL population (supplementary tables 2 and 3, Supplementary Material online). Therefore, we generated heterogeneous inbred family (HIF) lines from Cvi-0 × Col-0 RILs to fine-map LD2.1 and LD2.3 to respectively 70- and 34-kb intervals (supplementary fig. 2, Supplementary Material online). The two candidates *TIM22-1* and *TIM22-2* remained within the intervals.

To validate their causative role, we conducted a complementation cross between a heterozygous T-DNA mutant in *TIM22-2* in a Col-0 background (i.e., *TIM22-1* was nonfunctional) and the original HIF line in which *TIM22-1* was homozygous for the Col-0 genotype (i.e., also nonfunctional) and *TIM22-2* was heterozygous for Col-0/Cvi-0 (fig. 2*d*). Within 123 hybrids of the offspring, among the four possible allelic combinations at LD2.3, we did not find any hybrids combining a Cvi-0 and a T-DNA alleles at *TIM22-2* (fig. 2*d*), providing strong genetic evidence that the Cvi-0 allele cannot complement a knockout (T-DNA) allele at *TIM22-2* (in a background without other functional *TIM22* allele) and is thus nonfunctional.

Collectively, these segregation and complementation crosses show that the combination of different nonfunctional alleles of the *TIM22* copies leads to a drastic reduction of allelic combinations in offspring populations, and thus evidence the causative role of *TIM22* in this genetic incompatibility.

### Natural Modifiers Can Rescue Incompatible Allele Combinations

**Fig. 3.**
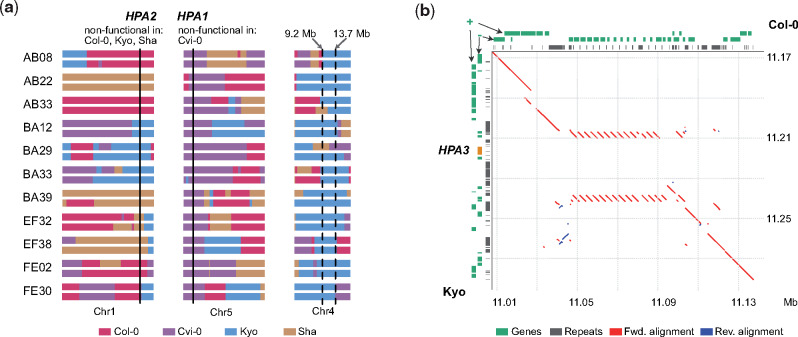
Incompatible allele combinations of *HPA* rescued by an additional gene copy. (*a*) Schematic of the genomes of 11 AMPRILs with presumably incompatible allele combinations of *HPA1* and *HPA2*. All these genotypes carry at least one Kyo allele at chr4: 9.2–13.7 Mb, suggesting that a Kyo allele in this region can rescue the incompatibility. (*b*) Sequence alignment around the *HPA3* locus on chromosome 4 between Col-0 and Kyo. The position of the third *HPA3* copy in Kyo is marked in orange. Red line, forward alignment; blue line, reverse alignment. Genes arrangement at forward (+) and reverse (−) strands, and repeat annotations are shown at the top (Col-0) or left (Kyo) axes.

Incompatible allele combinations can result in severe phenotypic defects, which lead to the reduction or the full absence of specific allele combinations. For example, the double homozygous nonfunctional allele combination of *HPA1/HPA2* results in embryo lethality (Bikard et al. 2009) and thereby wipes out all carriers of the incompatible allele combination. *HPA* encodes a histidinol-phosphate amino-transferase for the biosynthesis of histidine, an essential amino acid (Muralla et al. 2007). All eight AMPRIL founders except of Cvi-0 have a functional *HPA1* and a nonfunctional *HPA2* due to a premature stop codon or a hypermethylated promoter, whereas Cvi-0 carries a functional *HPA2* allele, but does not carry *HPA1* at all (supplementary table 5, Supplementary Material online). Unexpectedly, however, we did observe homozygous *HPA1/HPA2* incompatible allele combinations (*HPA2^−/−^HPA1^−/−^*) in 11 of the AMPRIL lines within the ABBA and EFFE subpopulations which were derived from Col-0, Cvi-0, Kyo, and Sha (supplementary tables 6–8, Supplementary Material online). Further analysis of these populations revealed an extremely high frequency of the Kyo allele on chromosome 4 (supplementary fig. 3, Supplementary Material online), making us recognize that the 11 AMPRIL lines with the incompatible allele combinations all carried at least one Kyo allele at chr4:9.2–13.7 Mb (fig. 3*a*). This suggested that Kyo might contain a modifying allele of the incompatibility complementing the lethal allele combination in this region (a similar feature was described as “conditional incompatibility” by Bikard et al. [2009] when analyzing crosses between Jea and Col-0). Indeed, when we checked the long-read assembly of the Kyo genome, we identified an additional *HPA* copy (hereafter named as *HPA3*) which colocated with the mapping interval at chr4:11.11 Mb (fig. 3*b*), whereas we could not find this allele in any of the other AMPRIL founder genomes.

### Mapping Modifiers of Incompatible Allele Combinations in Natural Populations

This analysis showed that the negative effects of incompatible allele combinations can be overcome if they are rescued by modifying alleles. Therefore, the virtual absence of functional alleles (among the known loci) of a duplicated gene could act as a molecular phenotype to map the location of additional (rescuing) alleles by genome-wide association (GWA) mapping even in natural populations.

**Fig. 4.**
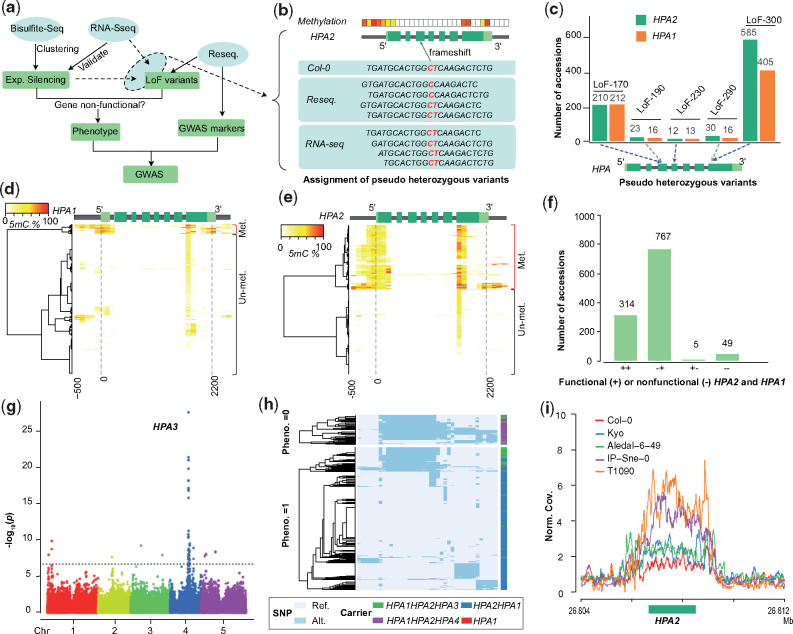
Mapping natural modifiers of genetic incompatibilities using GWAS. (*a*) Workflow for the identification of modifying alleles of genetic incompatibility using GWAS. (*b*) Schematic example to illustrate how genome-wide methylation and RNA-seq data are used to assign pseudoheterozygous variants to a specific gene copy despite the repetitive nature of the short-read alignments within duplicated genes. If a specific variation is present in DNA data, but absent in RNA data and one of the gene copies is methylated (i.e., likely expression silenced) the pseudoheterozygous variation is assigned to this (expression silenced) gene copy. Light green, untranslated region; green, coding region; gray, intron or gene up/down-stream. (*c*) Pseudoheterozygous LoF variants found in the short read alignments (of 1,135  *Arabidopsis thaliana* genomes; Alonso-Blanco et al. 2016) at *HPA2* and *HPA1*. LoF-170, LoF-230, and LoF-300 could be assigned to *HPA2* (using the procedure of (*b*)), whereas LoF-190 and LoF290 could not be assigned to either of the gene copies. (*d* and *e*) Hierarchical clustering of DNA methylation profiles in *HPA1* (*d*) and *HPA2* (*e*) based on the methylomes of 888 *A. thaliana* accessions from the 1001 Epigenome Project (Kawakatsu et al. 2016) (NCBI GEO accession number GSE43857). Methylation profiles calculated within 100-bp sliding windows from 500 bp upstream of the transcription start site to 300 bp downstream of the transcription end site. (*f*) The number of accessions with different functional copies of *HPA*2 and *HPA1* across 1,135 *A. thaliana* accessions. (*g*) Manhattan plot of a GWA using the absence of any functional *HPA* gene as phenotype. The most significantly associated locus reveals the region of *HPA3*. The dashed line indicates the significant threshold after multiple testing correction (−log_10_(*P*) = 6.68). (*h*) Two heatmaps of haplotype clustering (defined by the 39 significantly associated markers around the *HPA3* locus) shown for accessions with (below) or without (up) functional copies of *HPA2* or *HPA1*. (*i*) The normalized short read mapping coverage (Norm. Cov.: average mapping coverage at *HPA2* divided by average mapping coverage at whole genome) around *HPA2* based on read mappings against the Cvi-0 genome including only one *HPA* gene. (Data from five accessions are shown as examples to illustrate patterns of different *HPA* copies: Col-0: *HPA2HPA1*; Kyo and Aledal-6-49: *HPA2HPA1HPA3*; IP-Sne-0 and T1090: *HPA2HPA1HPA4*).

To do this, we searched for incompatible allele combinations (separately for all four incompatibilities) in each of the 1,135 accessions of the 1001 Genomes Project (Alonso-Blanco et al. 2016) and used these allele combinations as phenotype for a GWA (fig. 4). To define nonfunctional alleles, we used the resequencing data to search for LoF variations, and the methylome data from the 1001 Epigenomes Project (Kawakatsu et al. 2016) to identify methylated (silenced) promoters (fig. 4*a*, see Materials and Methods). Additionally, RNA-seq data were explored to distinguish pseudoheterozygous variants (which exist due to inaccurate short-read alignment at duplicated genes) and to check for gene silencing.

For the first incompatibility, in *HPA*, we found pseudoheterozygous LoF variations including two premature stop-codons and three frameshifts at both reference copies of *HPA* (fig. 4*c* and supplementary table 9, Supplementary Material online) due to the repetitiveness of the gene sequences. As some accessions did not show expression of *HPA2* most likely due to hypermethylation of their promoters, we tested which of the *HPA* alleles was present in the RNA-seq read data to assign the LoF to either of the *HPA* copies (fig. 4*b*). With this, we could assign one stop-codon gain (LoF-300) and two frameshifts (LoF-170, LoF-230) to *HPA2* because these LoF alleles were absent in the RNA-seq data in the accessions which lacked *HPA2* expression. Notably, the frameshift LoF-170 could be rescued by alternative splicing as observed in RNA-seq read mapping (supplementary fig. 4, Supplementary Material online). Furthermore, cytosine methylation profiles revealed hypermethylated promoters of *HPA2* in 340 accessions and of *HPA1* in 50 accessions (fig. 4*d* and *e*), suggesting gene silencing in these accessions, which was in agreement with the absence of pseudoheterozygous variants in the RNA-seq read data (fig. 4*b* and supplementary fig. 4, Supplementary Material online).

By combining the LoF variant genotyping and the methylation analyses, we found 767 accessions with a nonfunctional *HPA2* (*HPA2^−/−^ HPA1^+/+^*) allele and five accessions with a nonfunctional *HPA1* (*HPA2^+/+^HPA1^−/−^*) allele (fig. 4*f* and supplementary table 10, Supplementary Material online). In addition, 49 accessions did not feature any functional alleles (*HPA2^−/−^HPA1^−/−^*) and were expected to carry additional modifier(s) to complement the loss of functional copies. To find the locations of these modifiers, we used the absence of functional copies as phenotype (supplementary data 3, Supplementary Material online) to run a GWA under the mixed linear model using the SNP markers from the 1001 Genomes Project (www.1001genomes.org). This GWAS revealed a significantly associated region at chr4:11.00–11.15 Mb (fig. 4*g* and supplementary fig. 5, Supplementary Material online, alpha level of 0.05, Bonferroni correction), corresponding to the *HPA3* locus found in Kyo (chr4:11.11 Mb). Though other peaks in unlinked regions were present, these additional loci explained only a small proportion of the heritability.

Analyzing the haplotypes at the 39 significantly associated SNP markers at *HPA3* locus revealed a somewhat, but not entirely homogenous haplotype in many of the *HPA2^−/−^ HPA1^−^*^/^^*−*^ carriers (fig. 4*h*). To confirm that the modifying haplotype is still identical to the Kyo allele, we aligned the short reads of all accessions of the 1001 Genomes Project against the Kyo reference sequence and found that overall 162 accessions carried the *HPA3* allele (supplementary data 3, Supplementary Material online)*.* However, unexpectedly, the Kyo *HPA3* was only found in 13 of the 49 accessions without functional *HPA2* and *HPA1* alleles, suggesting the presence of two different modifiers at this locus on chromosome 4. To find more support for this, we ran a new GWA without the 162 *HPA3* (Kyo-like allele) carriers, which still led to a significantly associated locus at the region of *HPA3* (supplementary fig. 6, Supplementary Material online). Further short-read mappings of all 1,135 genomes against the Cvi-0 reference sequence (where only one copy of *HPA* exists) revealed the presence of (at least) two additional copies (in addition to *HPA1* and *HPA2*) in a total of 42 accessions including all the 36 accessions with the unknown rescuing alleles (fig. 4*i* and supplementary data 3, Supplementary Material online). Together this suggested that *HPA3* also rescues incompatible *HPA1* and *HPA2* allele combinations in natural populations and that the locus of *HPA3* contains an additional haplotype (hereafter named *HPA4*) which also rescues the incompatibility between *HPA1* and *HPA2*.

We continued to apply the same approach to the other three incompatibilities. For *TIM22*, 16 accessions of the 1001 Genome Project revealed nonfunctional allele combinations (supplementary table 11 and data 4, Supplementary Material online), again indicating the existence of modifying alleles. However, a GWAS using the presumably-incompatible allele combinations as molecular phenotype did not reveal only one, but numerous significantly associated loci (supplementary fig. 7, Supplementary Material online). This might be explained by the low number of incompatible allele carriers, which could affect the power of association mapping leading to false-positive associations. However, even though we could not locate the modifying allele, further analysis of read mapping coverage in *TIM22* revealed that all 16 accessions with nonfunctional *TIM22-1/TIM22-2* allele combinations carried at least one additional third copy of *TIM22*, whereas among all 1,119 other genomes of the 1001 Genomes Project only three genomes carried additional copies. This suggests that, like for *HPA*, known nonfunctional allele combinations of *TIM22* are in fact rescued by additional copies. Moreover, in ten of the 16 accessions, *TIM22-2* showed not only one but multiple additional copies (hereafter named as *TIM22-3*), which was further supported by the genome assembly of Ty-1 (https://genomevolution.org/CoGe/GenomeInfo.pl?gid=54584, unpublished), where we could find a cluster of four tandemly arranged *TIM22* gene copies at the *TIM22-2* locus.

**Fig. 5.**
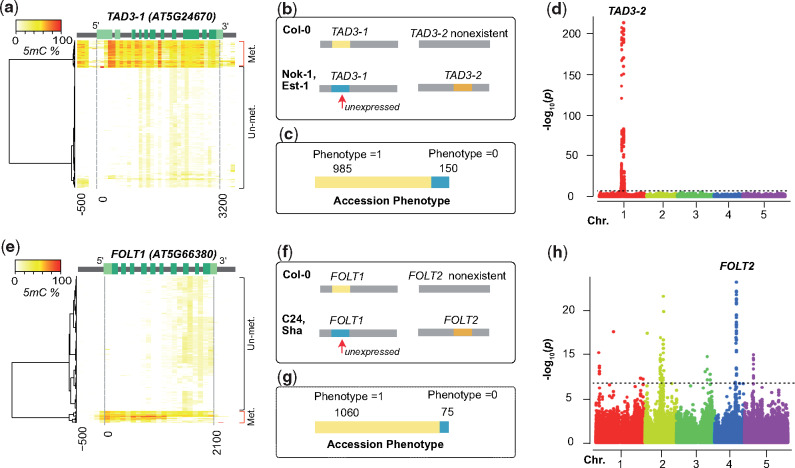
Mapping nonreference gene copies of incompatible alleles using GWAS. (*a* and *e*) Hierarchical clustering of cytosine methylation profiles in *TAD3-1* (*a*) and *FOLT1* (*e*) based on 888 *Arabidopsis thaliana* accessions from 1001 Epigenomes Project (Kawakatsu et al. 2016). The methylation profile was calculated based on 100-bp sliding windows from the 500 bp upstream of transcription start site to the 300 bp downstream of the transcription end site. Light green, UTR; green, coding region; gray, intron or up/down-stream. (*b* and *f*) The genetic incompatibilities introduced by *TAD3* (*b*) in hybrids between Col-0 and Nok-1/Est-1, and by *FOLT* (*f*) in hybrids between Col-0 and C24/Sha as shown previously (Durand et al. 2012; Agorio et al. 2017). (*c* and *g*) The number of accessions with functional (Phenotype = 1) or nonfunctional (Phenotype = 0) *TAD3-1* (*c*) and *FOLT1* (*g*) gene copies. (*d* and *h*) Manhattan plots of the GWA using the absence of functional *TAD3-1* (*d*) or *FOLT1* (*h*) gene copies as phenotype. The dashed line indicates the significance threshold after multiple testing correction (−log_10_*P *=* *6.68).

Because the reference sequence only contained one copy of *TAD3 (TAD3-1)* (Agorio et al. 2017) and *FOLT (FOLT1)* (Durand et al. 2012), we modified our GWA method and only used nonfunctional alleles at the reference gene as the phenotype to map modifiers for the two remaining incompatibilities (fig. 5 and supplementary fig. 8, Supplementary Material online). Due to this modification we would expect to map also the location of the duplicated genes as we had found them in the AMPRIL founders. For *TAD3*, an essential ortholog of the yeast tRNA Adenosine deaminase 3 (Gerber and Keller 1999), we did not find any accessions with LoF alleles, but we found 150 accessions with a hypermethylated promoter in *TAD3-1* similar to the methylated promoters found in Nok-1 and Est-1, which are known to be nonfunctional due to this methylated promotor region (Agorio et al. 2017) (fig. 5*a* and *b*). The GWA result revealed one significant peak (fig. 5*c* and *d*), which as expected colocated with the *TAD3-2* locus (Agorio et al. 2017). All the 150 accessions carried multiple additional copies of *TAD3-2* (supplementary data 5, Supplementary Material online), similar to Nok-1 and Est-1. This suggests that the expression silencing of *TAD3-1* is common in natural populations and that the rescue of this loss-of-function allele is generally mediated by additional gene copies at the *TAD3-2* locus as it was shown in the original description of this incompatibility (Agorio et al. 2017).

The last of the four genetic incompatibilities, introduced by *FOLT* encoding for a folate transporter, was previously discovered in hybrids from crosses between Col-0 × C24/Sha (Törjék et al. 2006; Simon et al. 2008; Durand et al. 2012). Col-0 has only one copy of *FOLT* (*FOLT1*) at chromosome 5, whereas the C24 and Sha have an additional copy, *FOLT2*, at chromosome 4 including some extra truncated copies near *FOLT2* (supplementary table 12, Supplementary Material online). In the earlier study, the truncated copies were shown to express siRNAs and activate the RNA-directed DNA methylation pathway to silence *FOLT1* in C24 and Sha (Durand et al. 2012), which resulted in a lethal allele combination in F2 hybrids between Col-0 (*FOLT1^+/+^*) and C24/Sha (*FOLT1^−/−^ FOLT2^+/+^*) (fig. 5*f*). Analyzing the accessions of the 1001 Genome Project for functional and nonfunctional alleles of *FOLT*, we found 75 accessions with methylated promoters of *FOLT1* likely leading to expression silencing (fig. 5*e*), which was supported by the lack of *FOLT1-*specific pseudoheterozygous SNPs in the RNA-seq data (supplementary fig. 9, Supplementary Material online). Besides providing evidence for the expression silencing of *FOLT1*, this also suggested the existence of additional *FOLT* gene copies in these 75 accessions. When we repeated our GWA approach to find these interacting loci of *FOLT1*, we found multiple significantly associated loci including one region corresponding to *FOLT2* (fig. 5*g* and *h*).

Further analyses of the *FOLT* gene copies using short read mapping against the C24 and Sha reference sequences revealed the presence of *FOLT2* in all the 75 accessions with methylated promoter of *FOLT1* and truncated copies of *FOLT2* genes in only 46 out of such 75 accessions (fig. 5*e* and supplementary table 13 and data 6, Supplementary Material online) suggesting that the methylation of the *FOLT1* promoter remains stable even after the truncated copies are segregated out. This is consistent to what was shown in successive generations of Sha × Col-0 RILs where the inducing locus was segregated away six generations ago (Durand et al. 2012).

Taken together, we found evidence that all four incompatible allele combinations, initially identified within an artificial intercross population, also occur in nature, and that the incompatible allele combinations of three of them were surprisingly common. For all incompatible allele combination carriers, we found evidence for the existence of additional copies, and could even map the locations of some of those using GWA.

### Geographic Distribution of Incompatible Allele Combinations

**Fig. 6.**
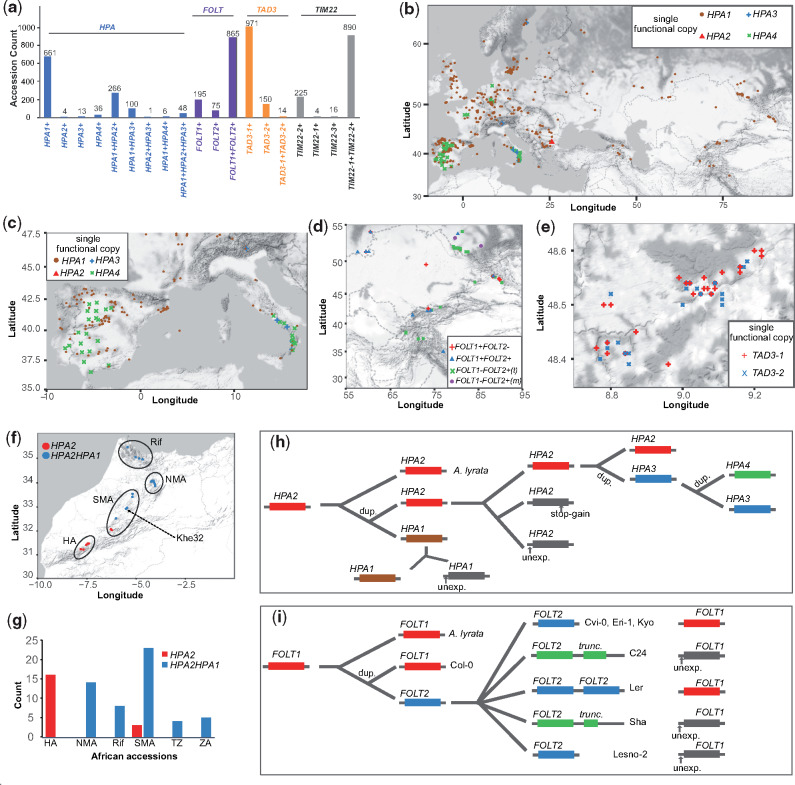
Distribution, origin, and evolution of incompatible alleles. (*a*) The number of accessions with different functional copies of *HPA*, *FOLT*, *TAD3*, and *TIM22* across 1,135 *Arabidopsis thaliana* accessions from the 1001 Genomes Project. +, functional. (*b*) Geographic distribution of accessions only with one functional copy of *HPA*. (*c*–*e*) Examples of geographically close accessions with only one functional copy of *HPA* in Southern Europe (*c*), *FOLT* in Central Asia (*d*), and *TAD3* in Germany (*e*). *FOLT1*-*FOLT2+*(t): accessions with a functional *FOLT2*, a truncated *FOLT2*, and an unexpressed *FOLT1*. *FOLT1*-*FOLT2+*(m): accessions with a functional *FOLT2* and an unexpressed *FOLT1*, but without truncated *FOLT2* copies. (*f* and *g*) Gene copies of *HPA* in African *A. thaliana* accessions. TZ, Tanzania; ZA, South Africa. (*h* and *i*) Schematic of a possible and parsimonious evolutionary history of gene duplication and nonfunctionalization of *HPA* (*h*) and *FOLT* (*i*). unexp., unexpressed; stop-gain, premature stop codon gained SNP; dup., gene duplication.

We next asked how prevalent the potential for incompatible allele combinations was within natural populations of *A. thaliana* by analyzing the presence of different haplotypes in different geographic regions. For this, we first analyzed the allele frequencies of different haplotypes (i.e., the different combinations of functional alleles within individual plants), which varied substantially across the accessions of the 1001 Genomes Project (fig. 6).

For example, among the accessions that carried only one functional copy of *HPA*, we found 661, 4, 13, and 36 accessions with a single functional copy of *HPA1*, *HPA2*, *HPA3*, or *HPA4*, respectively (fig. 6*a* and *b* and supplementary fig. 10, Supplementary Material online). Similarly, most accessions only featured one functional copy of *TAD3* including 971 accessions with only *TAD3-1* and 150 with only *TAD3-2* (fig. 6*a* and supplementary table 14 and supplementary fig. 11, Supplementary Material online). In contrast, most of the accessions included multiple functional alleles of *FOLT* as well as of *TIM22* (fig. 6*a*).

Genetic incompatibilities become effective in the offspring of accessions with incompatible alleles. In particular, the offspring of accessions with only one functional gene copy will lead to the highest reduction in fitness. We found numerous accessions with the potential to create incompatible allele combinations in their offspring in geographically close regions (fig. 6*c*–*e*). For example, among 30 accessions in the South of Italy we found nine accessions with only a functional *HPA1* closely located with 14 accessions with either a functional *HPA3* or a functional *HPA4* only (fig. 6*c*). Likewise, accessions with contrasting functional copies of *FOLT* and *TAD3* were collected from the same regions in Central Asia and Germany (fig. 6*d* and *e*) evidencing that incompatible alleles could segregate within the same populations.

Interestingly, within some of these contact zones of accessions with incompatible alleles, we also found accessions with multiple functional gene copies. For example, in South Italy there were seven of the 30 accessions that featured multiple functional copies of *HPA*, and similarly in Central Asia, we could find multiple accessions with two functional copies of *FOLT* in addition to the accessions with only one functional copy (fig. 6*d* and supplementary figs. 10–12, Supplementary Material online). In contrast, however, we only observed a few accessions with multiple functional alleles in the contact zone of incompatible *TAD3* alleles, most likely because only 14 such accessions were found in the entire set of accessions. Even though we could not find evidence that such contact zones are enriched for additional copies or that those additional copies would evolve in these regions, the presence of haplotypes with additional gene copies in these contact zones have the potential to mediate the gene flow between the haplotypes with the incompatible allele combinations.

### Origin and Evolution of Incompatible Alleles

To figure out when these incompatible alleles originated and how they evolved, we investigated their ancestral genotypes within African genotypes which likely represent the ancestral populations of the Eurasian accessions (Durvasula et al. 2017).

Although almost all (99.7%) of the Eurasian accessions had both *HPA2* and *HPA1* (either functional or not), we found that only 27% (20 out of 75) of the African accessions carried only *HPA2* (fig. 6*f* and *g*; supplementary data 3, Supplementary Material online). This suggested that *HPA2* was the ancestral copy of *HPA* which was further supported by the synteny alignment with the close relative *Arabidopsis lyrata* (Blevins et al. 2017), which only featured a single *HPA* gene in a region syntenic to *HPA2* and that the duplication events leading to *HPA1* happened early on in Africa (fig. 6*h*). One accession, Khe32, from Morocco carried *HPA1* along with the most frequent LoF variant LoF-300 in *HPA2* in the Eurasian accessions, suggesting that the first accession with only a functional *HPA1* possibly arose in North-West Africa and thereby suggests that the genetic incompatibility with *HPA2* carriers was already possible before the Eurasia colonization of *A. thaliana*.

In contrast, *HPA3* and *HPA4* only occur in Eurasian accessions, most likely indicating that the additional duplication events happened later (fig. 6*h*). Comparing the gene sequences of the *HPA* genes revealed that *HPA3* was duplicated from *HPA2* (supplementary fig. 13, Supplementary Material online), whereas *HPA4* might be a tandem duplicate of *HPA3* as it is likely located close to *HPA3*. Interestingly, *HPA3* and *HPA4* carriers segregated for the ancestral LoF-300 variant at *HPA2* (*HPA3*: 68 with and 94 without; *HPA4*: 10 with and 32 without, supplementary fig. 14, Supplementary Material online), suggesting free segregation of different *HPA* alleles. In striking contrast to this, the inactive alleles of *HPA1* (i.e., alleles with a nonfunctional *HPA1* sequence, but excluding the accessions with full deletion alleles as found in Cvi-0) were almost perfectly coupled (48 of 50) with inactive alleles of *HPA2*. Such carriers nearly all had *HPA4* (35) or *HPA3* (13) (supplementary fig. 15, Supplementary Material online) and were mainly located in the Iberian Peninsula and Southern Italy suggesting that the additional *HPA* copies were necessary to buffer the incompatibility and to allow the foundation of these populations (fig. 6*c*).

Unlike the incompatibility in *HPA*, genetic incompatibilities can also arise in recent population history. All African accessions only had the *TAD3-1* copy, whereas accessions with multiple copies of *TAD3* can only be observed in Eurasia (supplementary fig. 10, Supplementary Material online). Similarly, 38 of the African accessions only have *FOLT1*, whereas the other 37 accessions have both *FOLT1* and *FOLT2*, but none of the accessions featured a truncated copy of *FOLT2* (*FOLT2tr*), which is the mechanistic origin of the incompatible alleles at *FOLT1* and *FOLT2* (supplementary fig. 16 and data 6, Supplementary Material online). In contrast, within the Eurasian accession, we even found three different haplotypes of *FOLT2tr* within a total of 46 accessions based on short read alignments against the eight *A. thaliana* genomes (Jiao and Schneeberger 2020) (supplementary fig. 17 and data 6, Supplementary Material online). This together suggests that also the genetic incompatibility that is based on *FOLT2tr* has evolved recently after the migration *A. thaliana* to Eurasia (fig. 6*i*).

## Discussion

Although gene duplication provides genetic backup of essential genes, duplicated genes can also lead to incompatible allele combinations when the duplicated genes undergo reciprocal pseudofunctionalization in separate genomes (Lynch and Force 2000). Here, we first identified incompatible allele combinations of four duplicated gene pairs by integrating genetic and genomic information of a multiparental intercross population. Unexpectedly, when we searched the genomes of 1,135 accessions released by the 1001 Genomes Project (Alonso-Blanco et al. 2016), we identified many natural accessions with putatively incompatible allele combinations of these four genes and could elucidate the genetics of their incompatibilities and modifiers as they occur in natural populations using GWA. As duplicated genes are common in plant genomes (Lynch and Conery 2000; Maere et al. 2005; Panchy et al. 2016), duplicated gene based incompatibilities might be more common than typically anticipated, suggesting that our GWA approach could be further applied to search for more candidates in other genes.

Using multiomics data from hundreds of *A. thaliana* accessions, we found that incompatible alleles are surprisingly frequent in nature and also occur in sympatry, implying that the evolution of genetic incompatibilities does not require separated populations as proposed by BDM model (Bateson 1909; Dobzhansky 1937; Muller 1942). Moreover, incompatible allele combinations can persist over long periods as some of the alleles studied here may have originated before *A. thaliana* colonized Eurasia. This might be due to the inefficient elimination of nonfunctional alleles in a predominately selfing species (Bustamante et al. 2002). *Arabidopsis thaliana* wild populations have an outcrossing rate of around 1%, which however, can reach up to 15% in some outcrossing hotspots (Bakker et al. 2006; Bomblies et al. 2010), suggesting that the incompatible alleles might be recombined in the same individuals at some regions. Some recombined alleles (such as double homozygous recessive alleles) will be removed when no mutation of extrafunctional copy appears, which in turn can reduce the frequency of incompatible allele combinations.

As incompatibilities introduced by duplicated genes are likely caused by neutral mutations and genetic drift (Lynch and Force 2000), such neutral incompatibility alleles of duplicated genes are supposed to be purged even under low level of gene flow (Bank et al. 2012). Alternatively, when duplicated alleles are linked with other regions under constrains of selection, they could be also maintained during evolution. Taken together, the frequency and distribution of incompatible alleles at duplicated genes are dynamically affected by multiple factors including mating system, gene flow, and perhaps selection.

Although it remains unclear if incompatibilities in sympatry are common in other species as well, they have also been identified in *Minulus* species (Zuellig et al. 2018; Zuellig and Sweigart 2018). Using genotyping data from globally distributed individuals of other species such as rice where major resource have been generated (Huang et al. 2012; Wang et al. 2018) have the potential to reveal the frequency and distribution of incompatible duplicated alleles in these species in future (Mizuta et al. 2010; Yamagata et al. 2010; Nguyen et al. 2017).

Here, new gene copies arose either from distal duplications (in *HPA* and *FOLT*) or tandem duplications (in all four cases) and counteract incompatibilities. Even though additional copies reduce the frequency and impact of genetic incompatibilities, they could also increase the potential for more incompatible allele combinations. Subsequent subfunctionalization could be a way out of the trap of genetic incompatibilities, as there would be a selective pressure to keep both gene copies. Although we have assumed functional redundancy of all gene copies in this work, an extensive number of accessions shared the functional copies of both *FOLT* genes (865 of 1,135), which could indicate that these copies are not fully redundant and thereby limit the establishment of incompatible allele combinations. Thus, the incompatible alleles in sympatry may slow down neofunctionalization or pseudogenization of apparently redundant copies in multiple-copy carriers, and may partially explain why more duplicated genes are preserved than one would expect from theory (Lynch and Conery 2003; Maere et al. 2005).

Taken together, our work demonstrates that the potential for genetic incompatibilities due to duplicated essential genes is surprisingly high in nature. However, the effects of such incompatibilities are counteracted by additional gene copies, which undergo dynamic changes shaped by the recurrent events of gene duplication and nonfunctionalization during population history.

## Materials and Methods

### AMPRIL Construction

The eight *A. thaliana* accessions An-1, C24, Col-0, Cvi-0, Eri-1, Kyo, L*er*, and Sha were selected as the AMPRIL founders. We previously constructed a first version of AMPRIL population (AMPRIL I) including six RIL subpopulations (Huang et al. 2011). Here, AMPRIL I was extended with six additional subpopulations (called AMPRIL II) based on different pairwise intercrosses of the eight founders (fig. 1*a*). Each subpopulation contains approximately 90 individuals (supplementary data 1, Supplementary Material online). All plants were grown under the normal growth conditions in greenhouse at the MPI-PZ (Huang et al. 2011). DNA of 1,100 samples was extracted from the flower buds and prepared for RAD-seq sequencing (Baird et al. 2008).

### RAD-Seq Library Preparation and Sequencing

All plants were grown in the greenhouse. The total DNA from each of the 1,100 samples was extracted from the flower buds using DNeasy Plant Kit 96 (Qiagen) and eluted in 200 µl Elution buffer (EB). DNA of each genotype was isolated twice. Both genotype samples were pooled in 1.5 ml tube and the DNA was concentrated by isopropanol precipitation for 2 h at −20 °C. The samples were centrifuged at 12,000 × g for 5 min at 4 °C, the supernatant was removed, and the DNA pellet was washed with ice-cold 70% ethanol. Centrifugation was repeated, the supernatant was removed. Air-dried DNA was resuspended in a nuclease free water to 26 ng/µl and stored at −20 °C until use. RAD-seq sequencing libraries were prepared as described (Etter et al. 2011) with modifications. Per genotype, 500 ng DNA were digested with 10 units of CviQI (NEB, cutting site G’TAC) at 25 °C for 2 h. The number of expected cutting sites was estimated around 236,000 based on the Col-0 genome sequence. Cut DNA was purified using 96 DNA clean and concentrator kit (Zymo) a diluted in 25 µl EB. The 192 different (selected out of total 210 designed, supplementary data 1, Supplementary Material online) P1 adapters (200 nM) containing unique 12-bp barcodes were ligated by incubation with T4 ligase (NEB) at room temperature for 30 min and the reaction was terminated by 20 min at 65 °C. After 30 min at room temperature, 5 µl from each 192 P1-barcoded sample were combined in a 2 ml low bind tube. About 3 × 130 µl aliquots were transferred to fresh tubes and DNA was fragmented to average size of 500 bp using Covaris. Sheared DNA was purified using QiaMinElute columns (Qiagen), eluted in 10 µl EB, and the three samples were first pooled and then divided into two 15 µl samples that were run on 1% agarose gel. Regions of 300- to 500-bp fragments were dissected and DNA was isolated using MinElute gel extraction kit (Quiagen), eluted in 10 µl EB, the samples were pooled, DNA fragment ends were repaired using Quick Blunting kit (NEB), purified with QIAquick column (Qiagen), and eluted in 43 µl EB. 3′-deoxy-adenine overhangs were added using Klenow Fragment (NEB), the sample was purified with QIAquick column, eluted in 45 µl EB, the P2 adapter was ligated, the sample was purified with QIAquick column and eluted in 53 µl EB. To determine the library quality, 10 µl RAD library were PCR amplified (1: 98 °C 30 s; 2: 14× 98 °C 10 s, 67 °C 30 s, 68 °C 30 s; 3: 68 °C 5 min, 4: 4 °C hold) using NEB Next High-Fidelity master mix (NEB) in 25 µl reaction volume using RAD-Marker for/RAD-Marker rev primers (25 nM each) and 5 µl PCR product were loaded into 1% agarose gel next to the 1 µl RAD library template. If the PCR product smear was at least twice as intense as the template smear, the library was considered as of high quality and the amplification was repeated in 50 µl reaction volume. The product was cleaned using AMPure magnetic beads (Beckman Coulter) and dissolved in 20 µl EB. Finally, the sample was run on 1% agarose gel, the region of 300- to 500-bp fragments was cut out, DNA was isolated using MinElute Gel extraction kit (Qiagen), and eluted in 20 µl EB. The library was sequenced in an Illumina Hiseq2000 sequencing machine. Sequencing reads were demultiplexed according to the barcodes (supplementary data 1, Supplementary Material online).

### AMPRIL Genotyping

We used previously released whole-genome short read data (Jiao and Schneeberger 2020) to generate markers for genotyping the AMPRILs. We mapped the reads to the reference sequence and called SNPs using SHORE (version 0.9) (Ossowski et al. 2008) with default parameter settings. Only homozygous SNP calls were selected after removing SNP calls with low quality (quality < 30), in the repetitive regions or in regions with low mapping quality (quality < 30). The actual marker sets for each of the 12 subpopulations (ABBA, ACCA … GHHG) were selected based on respective parental genomes, exclusively selecting biallelic SNP markers. After excluding samples with replicates or too few sequencing reads, we performed genotyping on 992 AMPRILs using a Hidden Markov Model-based approach similar to the recently presented method for the reconstruction of genotypes derived from two parental genomes (Rowan et al. 2015) (for a detailed description, see supplementary note 1, Supplementary Material online).

### Identifying Genetic Incompatibilities Based on Duplicated Genes

Duplicated gene pairs were selected based on gene family clustering of protein-coding genes from all eight parental genomes using OrthoFinder (version 2.2.6) (Emms and Kelly 2015). We only selected interchromosome duplicated genes to avoid the effects of intrachromosome linkage (supplementary data 2, Supplementary Material online). For each duplicated gene pair, we required two copies in the reference sequence and at least one copy in one of the other genomes (DupGene2), for example, the genes *HPA* and *TIM22* (the reference Col-0 has two copies from two different chromosomes), or one copy in reference sequence and at least one copy on a different chromosome in at least one of the other parental genomes (DupGene1), for example, the gene *FOLT* (the reference Col-0 only has one copy, but Sha has two copies from two different chromosomes). We determined the genotypes (parental haplotypes) of each of the gene copies in each AMPRIL using the genotypes predicted in the middle of the respective reference gene, or in the case a gene was not present in the reference sequence—using the midpoint between the two closest flanking syntenic regions of the nonreference gene copy (based on synteny calculations from a previous study; Jiao and Schneeberger 2020). For example, the parental haplotype of one progeny at chromosome 5:24–27 Mb (where *FOLT2* is inside at 26.513–26.516 Mb) was homozygous Col-0, whereas it was homozygous for An-1 at chromosome 4:10–16 Mb (where *FOLT1* is inserted at 13.577 Mb according to synteny in the whole-genome alignment of the Col-0 and An-1 genomes). Therefore, the observed allele pair of *FOLT* in this progeny was *FOLT1*^Col-0/Col-0^*FOLT2*^An-1/An-1^.

We predicted candidate genetic incompatibilities using a two-step approach. In the first step, we performed χ^2^ tests (eq. 1) to check whether the frequency of allele pairs in duplicated genes was significantly distorted in any of the subpopulations or in any of two merged subpopulations (ABBA and EFFE or CDDC and GHHG which shared the same four founders, respectively).

(1) $$\chi^{2}=\;\sum_{i,j\;\in \{a,\;b,c,d\}}\frac{{\left(o_{ij}-e_{ij}\right)}^{2}}{e_{ij}}$$

Here, the *o_ij_* and *e_ij_* represent the observed and expected allele pair frequency of duplicated genes, respectively, and *a*, *b*, *c*, *d* represent the parental genotypes in each subpopulation. For example, the two copies of *Tim22*, *Tim22-1* and *Tim22-2*, are both present in the reference Col-0 genome. The observed and expected allele pair frequencies of *Tim22* in the subpopulation EGGE can be found in supplementary table 3, Supplementary Material online. There are 88 individuals in this subpopulation. If the genotype at these two loci in one individual genotype was aabb, the count of the allele combination “ab” was increased by two. However, if the genotype was heterozygous, for example, “abbc,” the counts of the allele combinations “ab” and “bc” were both increased by 1. The *P* value of the χ^2^ test is 2.5e-09, suggesting a significant distortion of allele combinations.

Additionally, we applied a modified χ^2^ test (eq. 2) for all the duplicated genes in the whole AMPRIL population by considering the effects of population structure.

(2) $$x^{2}=\;\sum_{i,j\;\in \{a,\;b,c,d,e,f,g,h\}}\frac{{\left(\sum o_{ij}-\;\sum e_{ij}\right)}^{2}}{\sum e_{ij}}.\;$$

Here, the *o_ij_* and *e_ij_* represent the observed and expected allele pair frequency of interchromosome duplicated genes, respectively, and *a*, *b*, *c*, *d, e, f, g*, and *h* represent all parental genotypes. The observed and expected allele pair frequencies in the whole population was the sum of observed ($\sum o_{ij}$) and expected ($\sum e_{ij}$) allele pair frequencies in each of the subpopulations.

The gene pairs with at least one significant segregation distortion in their observed allele combinations (FDR < 0.05) were kept for the next step. In this second step, we checked whether the respective gene copies contained LoF variation or methylated promoters (as described below). To address alternative splicing which is known to rescue LoF variation, we also checked the gene annotation within the parental genome assemblies to confirm the LoF. Only duplicated genes with confirmed LoF alleles in both copies of the duplication (in at least one of the parental genomes) were kept as candidates for genetic incompatibilities based on duplicated genes.

### Identification of LoF Variants in Candidate Genes

We mapped all whole-genome resequencing reads of 1,135 accessions from the 1001 Genomes Project (Alonso-Blanco et al. 2016), 75 accessions from Africa (Durvasula et al. 2017), and 118 accessions from China (Zou et al. 2017), to the Col-0 reference genome (TAIR10) (The Arabidopsis Genome Initiative 2000; Lamesch et al. 2012) using BWA (version 0.7.15) (Li and Durbin 2009) with the default parameter settings. SNPs and small indels were called using SAMtools (version 1.9) using default parameters (Li et al. 2009). Homozygous variants with mapping quality of more than 20 and with at least four reads aligned were kept. Pseudoheterozygous variants in *HPA* and *TIM22* were also recorded. The large insertion and deletions in the 40-kb extended genic region of focal genes were predicted using Pindel (version 0.2.5) (Ye et al. 2009) with parameter settings “-T 1 -x 5 -k -r -j” and Delly (version 0.8.1) (Rausch et al. 2012) with parameter settings “delly call -q 20 -r 20 -n -u 20 –g.” The functional effects of these variations were annotated using SnpEff (version 4.3p) (Cingolani et al. 2012) using the default parameter settings. The LoF effects include loss of start codon, loss of stop codon, gain of premature stop codon, damage of splicing acceptor or donor sites, frameshift, and CDS loss.

### Clustering of Cytosine Methylation Profile in Gene Promoter

Cytosine methylation data (the tab separated file of methylated cytosine positions) of 1,211 samples from the 1001 Epigenomes Project (Kawakatsu et al. 2016) were downloaded from NCBI (927 samples under GEO accession GSE43857 and 284 samples under GEO accession GSE54292). After removing the redundant samples, 888 and 161 data sets from GSE43857 and GSE54292, respectively, were retained. For each sample, we calculated the percentage of methylated cytosines in CG, CHG, and CHH contexts from 500 bp upstream of the transcription start sites to 300 bp downstream of the transcription termination sites of each candidate gene in 100-bp nonoverlapping sliding windows. These methylation profiles were hierarchically clustered using the hclust function implemented in R (version 3.5.1). The pairwise, Euclidean distances between all methylation profiles were calculated and Ward’s method was used to cluster the samples into two groups (hypermethylated and unmethylated). This clustering was performed for the samples of GSE43857 and GSE54292 separately as these two data sets were processed in two studies with different pipelines (Dubin et al. 2015; Kawakatsu et al. 2016). The heatmap of methylation patterns was drawn in R using the heatmap.2 function.

### Analysis of DNA Methylation within the Genomes of the AMPRIL Founders

For six accessions (Col-0, An-1, C24, Cvi-0, Ler, Kyo), we downloaded the whole-genome DNA methylation data from NCBI from the 1001 Epigenomes Project (Kawakatsu et al. 2016) (GSE43857). For Eri-1 and Sha, DNA methylation data were generated using whole-genome bisulfite sequencing by the Max Planck Genome center. DNA was extracted from plants grown in the greenhouse under standard conditions using the Qiagen DNEasy Plant Mini Kit (Qiagen, Germany) and a sequencing library was prepared using the NEXTflex Bisulfite Library Prep Kit. This library was sequenced on an Illumina HiSeq2000 machine. Sequencing reads were aligned the reference sequence using Bismark (version 0.20.0) (Krueger and Andrews 2011) with these parameters “-q –bowtie2 -N 1 -L 24 -p 20.” The cytosine methylation profiles in candidate genes were calculated using the same sliding window method as described above. The cytosine methylation profiles together with the profiles from GSE43857 were then clustered again with the same clustering method as described above.

### Mapping Modifiers of Incompatible Alleles Using GWA

We predicted the presence and absence of functional copies of duplicated genes (*HPA*, *TIM22*) in each of 1,135 accessions from the 1001 Genomes Project according to the annotations of LoF variations and the clustering of cytosine methylation profiles in promoters (supplementary data 3–6, Supplementary Material online). For accessions without available methylation sequencing data, we assume the focal genes are expressed. The presence or absence of any functional copies of the reference genes were used as binary phenotype (presence: 1, absence: 0). For the association, we selected 238,166 high-quality SNP markers (minor allele frequency >0.05 and missing rate <0.1) from 1001 Genomes Project and imputed missing alleles with IMPUTE2 (Howie et al. 2009; Howie et al. 2012). An in-house R script (https://github.com/schneebergerlab/AMPRIL-GI/blob/master/GWAS/gwas.LM.multipro.r) implementing the mixed linear model with correction of kinship bias was used to perform the GWA (Bonferroni correction, *P* < 0.05).

### Copy Number Variation Analysis

To test for the existence of *HPA3* in a genome, we mapped whole-genome short reads of 1,135 accessions from 1001 Genomes Project (Alonso-Blanco et al. 2016), of 75 accessions from Africa (Durvasula et al. 2017) and of 118 accessions from China (Zou et al. 2017) to the Kyo genome assembly (Jiao and Schneeberger 2020) using BWA (version 0.7.15) with default parameters. Accessions with an average mapping coverage ≥5 along the *HPA3* region and duplicated region breakpoints (identified based on the sequence alignment against Col-0 genome using SyRI; Goel et al. 2019; version 1.0; with default parameters) were considered as *HPA3* carriers.

The copy number of duplicated genes was estimated by the ratio between the average mapping coverage within the focal gene and the average mapping coverage across the whole genome. To estimate the copy number of *HPA*, we mapped the short reads to the Cvi-0 genome assembly using BWA (version 0.7.15) with default parameters as the Cvi-0 genome only has one copy of *HPA*. For *TAD3*, *FOLT*, and *TIM22*, the copy number was predicted based on short reads mapping against the reference genome where both *TAD3* and *FOLT* only have one copy. For the *TIM22*, copy number was estimated based on the average mapping coverage at both *TIM22-1* and *TIM22-2*.

### Fine-Mapping of Incompatible Allele at LD2 in Cvi-0 × Col-0 RIL

After the observation that it was not possible to obtain a RIL homozygous for the Col-0 allele at LD2.1 while homozygous for the Cvi-0 allele at LD2.3 (Simon et al. 2008), we derived two distinct heterogeneous inbred families (HIF) from two segregating RILs from this population (supplementary fig. 2, Supplementary Material online). 8RV467 is segregating for a region largely encompassing LD2.1 while fixed Cvi-0 at LD2.3. 8RV408 is segregating for LD2.3 while fixed Col-0 at LD2.1. In each derived HIF family, it is again not possible to fix the incompatible allele combination, so the families were used to exclude intervals that do not contribute to the incompatible interaction. Two rounds of fine-mapping were conducted by genotypically screening increasing populations of descendants (at the seedling stage) for recombinants in the interval. Gradually, the causal interval is reduced and delineated by markers exploiting known SNPs and indels in the Cvi-0 sequence.

### Segregation of T-DNA Mutant Line at Gene *TIM22-1* and *TIM22-2*

T-DNA lines GABI_848H04 segregates for an insertion in *TIM22-1*, whereas GABI_626H10 segregates for an insertion in *TIM22-2*, both in a Col-0 background. Descendant was screened genotypically at the seedling stage to characterize the segregation of the T-DNA insertion allele.

### Complementation Cross to Validate the Incompatible Alleles of *TIM22*

A LD2.3 HIF line (8HV408-Het) was crossed with a GABI_626H10 line, both at the heterozygous state, in order to segregate potentially for all four possible hybrid allelic combinations at LD2.3, while maintaining a fixed Col-0 allele at LD2.1. The F1 descendant (123 individuals, from both cross directions) was screened genotypically at the seedling stage for the presence/absence of both the T-DNA insertion and the Cvi-0 allele.

### Code Availability

Custom code used in this study can be freely accessed at https://github.com/schneebergerlab/AMPRIL-GI.

### Seed Availability

We are currently preparing seeds of new AMPRIL populations for submission to NASC (http://arabidopsis.info/). Note heterozygous regions as reported in the genotype data might be fixed for one of the alleles in the requested seeds.

## Supplementary Material

Supplementary data are available at *Molecular Biology and Evolution* online.

## Supplementary Material

msaa306_Supplementary_Data

## References

[msaa306-B1] Ackermann M , Beyer A. 2012. Systematic detection of epistatic interactions based on allele pair frequencies. PLoS Genet. 8(2):e1002463. 22346757 10.1371/journal.pgen.1002463 PMC3276547

[msaa306-B2] Agorio A , Durand S, Fiume E, Brousse C, Gy I, Simon M, Anava S, Rechavi O, Loudet O, Camilleri C, et al 2017. An Arabidopsis natural epiallele maintained by a feed-forward silencing loop between histone and DNA. PLoS Genet. 13(1):e1006551. 28060933 10.1371/journal.pgen.1006551 PMC5257005

[msaa306-B3] Alonso-Blanco C , Andrade J, Becker C, Bemm F, Bergelson J, Borgwardt KMM, Cao J, Chae E, Dezwaan TMM, Ding W, et al 2016. 1,135 genomes reveal the global pattern of polymorphism in *Arabidopsis thaliana*. Cell 166(2):481–491. 27293186 10.1016/j.cell.2016.05.063 PMC4949382

[msaa306-B4] Arabidopsis Genome Initiative. 2000. Analysis of the genome sequence of the flowering plant *Arabidopsis thaliana*. Nature 408:796–815. 11130711 10.1038/35048692

[msaa306-B5] Baird NA , Etter PD, Atwood TS, Currey MC, Shiver AL, Lewis ZA, Selker EU, Cresko WA, Johnson EA. 2008. Rapid SNP discovery and genetic mapping using sequenced RAD markers. PLoS One 3(10):e3376–e3377. 18852878 10.1371/journal.pone.0003376 PMC2557064

[msaa306-B6] Bakker EG , Stahl EA, Toomajian C, Nordborg M, Kreitman M, Bergelson J. 2006. Distribution of genetic variation within and among local populations of *Arabidopsis thaliana* over its species range. Mol Ecol. 15(5):1405–1418. 16626462 10.1111/j.1365-294X.2006.02884.x

[msaa306-B7] Bank C , Bürger R, Hermisson J. 2012. The limits to parapatric speciation: Dobzhansky-Muller incompatibilities in a continent-Island model. Genetics 191(3):845–863. 22542972 10.1534/genetics.111.137513 PMC3389979

[msaa306-B8] Bateson W. 1909. Heredity and variation in modern lights. In: Seward AC, editor. Darwin and modern science: essays in Commemoration of the Centenary of the Birth of Charles Darwin and of the Fiftieth Anniversary of the Publication of the Origin of Species. Cambridge: Cambridge University Press. p. 85–101.

[msaa306-B9] Bikard D , Patel D, Le Mette C, Giorgi V, Camilleri C, Bennett MJ, Loudet O. 2009. Divergent evolution of duplicate genes leads to genetic incompatibilities within *A. thaliana*. Science 323(5914):623–626. 19179528 10.1126/science.1165917

[msaa306-B10] Blevins T , Wang J, Pflieger D, Pontvianne F, Pikaard CS. 2017. Hybrid incompatibility caused by an epiallele. Proc Natl Acad Sci U S A. 114(14):3702–3707. 28270603 10.1073/pnas.1700368114 PMC5389275

[msaa306-B11] Bomblies K , Yant L, Laitinen RA, Kim ST, Hollister JD, Warthmann N, Fitz J, Weigel D. 2010. Local-scale patterns of genetic variability, outcrossing, and spatial structure in natural stands of *Arabidopsis thaliana*. PLoS Genet. 6(3):e1000890–e1000914. 20361058 10.1371/journal.pgen.1000890 PMC2845663

[msaa306-B12] Bustamante CD , Nielsen R, Sawyer SA, Olsen KM, Purugganan MD, Hartl DL. 2002. The cost of inbreeding in *Arabidopsis*. Nature 416(6880):531–534. 11932744 10.1038/416531a

[msaa306-B13] Cingolani P , Platts A, Wang LL, Coon M, Nguyen T, Wang L, Land SJ, Lu X, Ruden DM. 2012. A program for annotating and predicting the effects of single nucleotide polymorphisms, SnpEff: SNPs in the genome of *Drosophila melanogaster* strain w1118; iso-2; iso-3. Fly 6(2):80–92. 22728672 10.4161/fly.19695 PMC3679285

[msaa306-B14] Conant GC , Wolfe KH. 2008. Turning a hobby into a job: how duplicated genes find new functions. Nat Rev Genet. 9(12):938–950. 19015656 10.1038/nrg2482

[msaa306-B15] Corbett-Detig RB , Zhou J, Clark AG, Hartl DL, Ayroles JF. 2013. Genetic incompatibilities are widespread within species. Nature 504(7478):135–137. 24196712 10.1038/nature12678 PMC4844467

[msaa306-B16] Dobzhansky T. 1937. Genetics and the origin of species. New York: Columbia University Press.

[msaa306-B17] Dubin MJ , Zhang P, Meng D, Remigereau MS, Osborne EJ, Casale FP, Drewe P, Kahles A, Jean G, Vilhjálmsson B, et al 2015. DNA methylation in Arabidopsis has a genetic basis and shows evidence of local adaptation. Elife. 4:1–23. 10.7554/eLife.05255 PMC4413256 25939354

[msaa306-B18] Durand S , Bouché N, Perez Strand E, Loudet O, Camilleri C. 2012. Rapid establishment of genetic incompatibility through natural epigenetic variation. Curr. Biol. 22(4):326–331. 22285031 10.1016/j.cub.2011.12.054

[msaa306-B19] Durvasula A , Fulgione A, Gutaker RM, Alacakaptan SI, Flood PJ, Neto C, Tsuchimatsu T, Burbano HA, Picó FX, Alonso-Blanco C, et al 2017. African genomes illuminate the early history and transition to selfing in *Arabidopsis thaliana*. Proc Natl Acad Sci U S A. 114(20):5213–5218. 28473417 10.1073/pnas.1616736114 PMC5441814

[msaa306-B20] Emms DM , Kelly S. 2015. OrthoFinder: solving fundamental biases in whole genome comparisons dramatically improves orthogroup inference accuracy. Genome Biol. 16(1):157. 26243257 10.1186/s13059-015-0721-2 PMC4531804

[msaa306-B21] Etter PD , Bassham S, Hohenlohe PA, Johnson EA, Cresko WA. 2011. SNP discovery and genotyping for evolutionary genetics using RAD sequencing BT. In: Orgogozo V, Rockman MV, editors. Molecular methods for evolutionary genetics. Totowa (NJ): Humana Press. p. 157–178. 10.1007/978-1-61779-228-1_9 PMC3658458 22065437

[msaa306-B22] Fishman L , Sweigart AL. 2018. When two rights make a wrong: the evolutionary genetics of plant hybrid incompatibilities. Annu Rev Plant Biol. 69(1):707–717.25. 29505737 10.1146/annurev-arplant-042817-040113

[msaa306-B23] Gerber AP , Keller W. 1999. An adenosine deaminase that generates inosine at the wobble position of tRNAs. Science 286(5442):1146–1149. 10550050 10.1126/science.286.5442.1146

[msaa306-B24] Goel M , Sun H, Jiao W-B, Schneeberger K. 2019. SyRI: finding genomic rearrangements and local sequence differences from whole-genome assemblies. Genome Biol. 20(1):277. 31842948 10.1186/s13059-019-1911-0 PMC6913012

[msaa306-B25] Howie B , Fuchsberger C, Stephens M, Marchini J, Abecasis GR. 2012. Fast and accurate genotype imputation in genome-wide association studies through pre-phasing. Nat Genet. 44(8):955–959. 22820512 10.1038/ng.2354 PMC3696580

[msaa306-B26] Howie BN , Donnelly P, Marchini J. 2009. A flexible and accurate genotype imputation method for the next generation of genome-wide association studies. PLoS Genet. 5(6):e1000529. 19543373 10.1371/journal.pgen.1000529 PMC2689936

[msaa306-B27] Huang X , Kurata N, Wei X, Wang ZX, Wang A, Zhao Q, Zhao Y, Liu K, Lu H, Li W, et al 2012. A map of rice genome variation reveals the origin of cultivated rice. Nature 490(7421):497–501. 23034647 10.1038/nature11532 PMC7518720

[msaa306-B28] Huang X , Paulo M-J, Boer M, Effgen S, Keizer P, Koornneef M, van Eeuwijk FA. 2011. Analysis of natural allelic variation in *Arabidopsis* using a multiparent recombinant inbred line population. Proc Natl Acad Sci U S A. 108(11):4488–4493. 21368205 10.1073/pnas.1100465108 PMC3060268

[msaa306-B29] Jiao W , Schneeberger K. 2020. Chromosome-level assemblies of multiple Arabidopsis genomes reveal hotspots of rearrangements with altered evolutionary dynamics. Nat Commun. 11(1):989. 32080174 10.1038/s41467-020-14779-y PMC7033125

[msaa306-B30] Kawakatsu T , Huang S, Shan C, Jupe F, Sasaki E, Schmitz RJJ, Urich MA, Castanon R, Nery JRR, Barragan C, He Y, et al 2016. Epigenomic diversity in a global collection of *Arabidopsis thaliana* accessions. Cell 166(2):492–506. 27419873 10.1016/j.cell.2016.06.044 PMC5172462

[msaa306-B31] Kondrashov FA. 2012. Gene duplication as a mechanism of genomic adaptation to a changing environment. Proc R Soc B. 279(1749):5048–5057. 10.1098/rspb.2012.1108 PMC3497230 22977152

[msaa306-B32] Krueger F , Andrews SR. 2011. Bismark: a flexible aligner and methylation caller for bisulfite-Seq applications. Bioinformatics 27(11):1571–1572. 21493656 10.1093/bioinformatics/btr167 PMC3102221

[msaa306-B33] Lamesch P , Berardini TZ, Li D, Swarbreck D, Wilks C, Sasidharan R, Muller R, Dreher K, Alexander DL, Garcia-Hernandez M, et al 2012. The Arabidopsis Information Resource (TAIR): Improved gene annotation and new tools. Nucleic Acids Res. 40:D1202–D1210. 10.1093/nar/gkr1090 PMC3245047 22140109

[msaa306-B34] Li H , Durbin R. 2009. Fast and accurate short read alignment with Burrows-Wheeler transform. Bioinformatics 25(14):1754–1760. 19451168 10.1093/bioinformatics/btp324 PMC2705234

[msaa306-B35] Li H , Handsaker B, Wysoker A, Fennell T, Ruan J, Homer N, Marth G, Abecasis G, Durbin R, 1000 Genome Project Data Processing Subgroup. 2009. The Sequence Alignment/Map format and SAMtools. Bioinformatics 25(16):2078–2079. 19505943 10.1093/bioinformatics/btp352 PMC2723002

[msaa306-B36] Lynch M , Conery JS. 2000. The evolutionary fate and consequences of duplicate genes. Science 290(5494):1151–1155. 11073452 10.1126/science.290.5494.1151

[msaa306-B37] Lynch M , Conery JS. 2003. The evolutionary demography of duplicate genes. J Struct Funct Genomics. 3(1/4):35–44. 12836683

[msaa306-B38] Lynch M , Force AG. 2000. The origin of interspecific genomic incompatibility via gene duplication. Am Nat. 156(6):590–605. 29592543 10.1086/316992

[msaa306-B39] Maere S , De Bodt S, Raes J, Casneuf T, Van Montagu M, Kuiper M, Van De Peer Y. 2005. Modeling gene and genome duplications in eukaryotes. Proc Natl Acad Sci U S A. 102(15):5454–5459. 15800040 10.1073/pnas.0501102102 PMC556253

[msaa306-B40] Maheshwari S , Barbash DA. 2011. The genetics of hybrid incompatibilities. Annu Rev Genet. 45(1):331–355. 21910629 10.1146/annurev-genet-110410-132514

[msaa306-B41] Mizuta Y , Harushima Y, Kurata N. 2010. Rice pollen hybrid incompatibility caused by reciprocal gene loss of duplicated genes. Proc Natl Acad Sci U S A. 107(47):20417–20422. 21048083 10.1073/pnas.1003124107 PMC2996679

[msaa306-B42] Muller HJ. 1942. Isolating mechanisms, evolution and temperature. Biol Symp. 6:71–125.

[msaa306-B43] Muralla R , Sweeney C, Stepansky A, Leustek T, Meinke D. 2007. Genetic dissection of histidine biosynthesis in *Arabidopsis*. Plant Physiol. 144(2):890–903. 17434988 10.1104/pp.107.096511 PMC1914156

[msaa306-B44] Murcha MW , Elhafez D, Lister R, Tonti-Filippini J, Baumgartner M, Philippar K, Carrie C, Mokranjac D, Soll J, Whelan J. 2007. Characterization of the preprotein and amino acid transporter gene family in *Arabidopsis*. Plant Physiol. 143(1):199–212. 17098851 10.1104/pp.106.090688 PMC1761978

[msaa306-B45] Nguyen GN , Yamagata Y, Shigematsu Y, Watanabe M, Miyazaki Y. 2017. Duplication and loss of function of genes encoding RNA polymerase III subunit C4 causes hybrid incompatibility in rice. G3 (Bethesda) 7:2565–2575. 28592558 10.1534/g3.117.043943 PMC5555463

[msaa306-B46] Ossowski S , Schneeberger K, Clark RM, Lanz C, Warthmann N, Weigel D. 2008. Sequencing of natural strains of *Arabidopsis thaliana* with short reads. Genome Res. 10.1101/gr.080200.108 PMC2593571 18818371

[msaa306-B47] Panchy N , Lehti-Shiu M, Shiu S-H. 2016. Evolution of gene duplication in plants. Plant Physiol. 171(4):2294–2316. 27288366 10.1104/pp.16.00523 PMC4972278

[msaa306-B48] Rausch T , Zichner T, Schlattl A, Stütz AM, Benes V, Korbel JO. 2012. DELLY: Structural variant discovery by integrated paired-end and split-read analysis. Bioinformatics 28(18):i333–339. 22962449 10.1093/bioinformatics/bts378 PMC3436805

[msaa306-B49] Rowan BA , Patel V, Weigel D, Schneeberger K. 2015. Rapid and inexpensive whole-genome genotyping-by-sequencing for crossover localization and fine-scale genetic mapping. G3 (Bethesda) 5:385–398. 25585881 10.1534/g3.114.016501 PMC4349092

[msaa306-B50] Simon M , Loudet O, Durand S, Bérard A, Brunel D, Sennesal FX, Durand-Tardif M, Pelletier G, Camilleri C. 2008. Quantitative trait loci mapping in five new large recombinant inbred line populations of *Arabidopsis thaliana* genotyped with consensus single-nucleotide polymorphism markers. Genetics 178(4):2253–2264. 18430947 10.1534/genetics.107.083899 PMC2323813

[msaa306-B51] Törjék O , Witucka-Wall H, Meyer RC, Von Korff M, Kusterer B, Rautengarten C, Altmann T. 2006. Segregation distortion in Arabidopsis C24/Col-0 and Col-0/C24 recombinant inbred line populations is due to reduced fertility caused by epistatic interaction of two loci. Theor Appl Genet. 113(8):1551–1561. 16988816 10.1007/s00122-006-0402-3

[msaa306-B52] Vaid N , Laitinen RAE. 2019. Diverse paths to hybrid incompatibility in *Arabidopsis*. Plant J. 97(1):199–213. 30098060 10.1111/tpj.14061

[msaa306-B53] Wang W , Mauleon R, Hu Z, Chebotarov D, Tai S, Wu Z, Li M, Zheng T, Fuentes RR, Zhang F, et al 2018. Genomic variation in 3,010 diverse accessions of Asian cultivated rice. Nature 557(7703):43–49. 29695866 10.1038/s41586-018-0063-9 PMC6784863

[msaa306-B54] Yamagata Y , Yamamoto E, Aya K, Win KT, Doi K, Sobrizal Ito T, Kanamori H, Wu J, Matsumoto T, et al 2010. Mitochondrial gene in the nuclear genome induces reproductive barrier in rice. Proc Natl Acad Sci U S A. 107:1494–1499. 20080642 10.1073/pnas.0908283107 PMC2824375

[msaa306-B55] Ye K , Schulz MH, Long Q, Apweiler R, Ning Z. 2009. Pindel: a pattern growth approach to detect break points of large deletions and medium sized insertions from paired-end short reads. Bioinformatics 25(21):2865–2871. 19561018 10.1093/bioinformatics/btp394 PMC2781750

[msaa306-B56] Zou Y-P , Hou X-H, Wu Q, Chen J-F, Li Z-W, Han T-S, Niu X-M, Yang L, Xu Y-C, Zhang J, et al 2017. Adaptation of *Arabidopsis thaliana* to the Yangtze River basin. Genome Biol. 18(1):239. 29284515 10.1186/s13059-017-1378-9 PMC5745794

[msaa306-B57] Zuellig MP , Sweigart AL. 2018. A two-locus hybrid incompatibility is widespread, polymorphic, and active in natural populations of *Mimulus*. Evolution 72(11):2394–2405. 30194757 10.1111/evo.13596 PMC6527092

[msaa306-B58] Zuellig MP , Sweigart AL. 2018. Gene duplicates cause hybrid lethality between sympatric species of *Mimulus*. PLoS Genet. 14(4):e1007130. 29649209 10.1371/journal.pgen.1007130 PMC5896889

