# Supplementary material for: The Evolutionary Dynamics of Genetic Incompatibilities Introduced by Duplicated Genes in Arabidopsis thaliana
Source: Mol Biol Evol. 2020 Nov 28;38(4):1225–40. doi: 10.1093/molbev/msaa306 (PMC8042742; doi:10.1093/molbev/msaa306)
Supplement: msaa306_Supplementary_Data [file msaa306_supplementary_data.zip › Jiao_et_al.Supplementary.v2.pdf]

Supplementary Data 1. Summary of RAD-seq data of AMPRIL population

Supplementary Data 2. List of inter-chromosome duplicated genes.

Supplementary Data 3. Loss-of-function variation, promoter methylation profile clustering and copy number of *HPA* across 1,135 *A. thaliana* accessions from the 1001 Genomes Project, 75 African accessions and 118 Chinese accessions.

Supplementary Data 4. Loss-of-function variation, promoter methylation profile clustering and copy number of *TIM22* across 1,135 *A. thaliana* accessions from the 1001 Genomes Project, 75 African accessions and 118 Chinese accessions.

Supplementary Data 5. Loss-of-function variation, promoter methylation profile clustering and copy number of *TAD3* across 1,135 *A. thaliana* accessions from the 1001 Genomes Project, 75 African accessions and 118 Chinese accessions.

Supplementary Data 6. Loss-of-function variation, promoter methylation profile clustering and copy number of *FOLT* across 1,135 *A. thaliana* accessions from the 1001 Genomes Project, 75 African accessions and 118 Chinese accessions.

Supplementary Note 1

Pre-genotyping

The pre-genotyping was performed in each subpopulation separately. For each sub-population, SNP markers were selected based on the SNP markers identification between the respective parental genomes. Each sample was pre-genotyped at each of the SNP marker positions based on the number of aligned short reads under the equation 1 (Eq. 1). The probabilities of two homozygous paternal genotypes (x_1, x_2) and heterozygous genotype $f(a, b)$ were estimated as

$$x_1 = \frac{(a+b)!}{a! * b!} * 0.99^a * 0.01^b$$

$$x_2 = \frac{(a+b)!}{a! * b!} * 0.99^b * 0.01^a$$

$$f(a,b) = \frac{(a+b)!}{a! * b!} * p^a * p^b,\ p = 0.5 \qquad \text{(Eq.1)}$$

Here, the *a* and *b* represent the read counts for the reference and alternative allele. 0.01 is the assumed error rate for an allele as observed in the short-read alignments. This step of pre-genotyping was implemented in our previously released tool TIGER (Rowan et al. 2015).

Two stage Hidden-Markov-Models

To translate the raw genotypes to identity-by-decent haplotype assignments, two different HMMs were developed for the homozygous (HMM-1) and the heterozygous (HMM-2) part of the genome. In HMM-1, five hidden states were defined and labelled as A, B, C, D (which represent the four parents of a subpopulation) and Z (which represents heterozygous regions). The emission states represent the possible observation values, which are from the pre-genotyping step. Regions labeled as Z were further analyzed by the HMM-2. The HMM-2 contains ten hidden states, where each hidden state has ten emission states. The ten hidden states represent all possible allele combinations (four homozygous and six heterozygous genotypes) from the offspring of hybrids of four parents. To reduce the errors of genotyping, the HMM-2 only considered SNPs markers, which were unique for one of the parents at that marker position. Besides, to reduce errors at the transitions between heterozygous and homozygous regions, in addition to the markers in the Z regions, 100 markers within the flanking homozygous regions were considered.

Simulations and training of the HMM

To train and validate our method, we firstly simulated a multiparent population with the same crossing scheme as the AMPRIL subpopulations including 5,000 samples. Three different average sequencing coverage rates (0.1x, 1x, and 10x) were simulated and 1.2 million markers

were generated. The recombination rate was simulated using an extended version of the tool Pop-seq (Salomé et al. 2012; James et al. 2013) (http://sourceforge.net/projects/popseq). Firstly, the probability matrix for the transition and emission was estimated using a supervised learning strategy by selecting 1,000 randomly simulated samples with an average sequencing coverage rate of 1x. Then, we applied the resulting HMM to ten randomly sequenced samples and changed slightly the emission probabilities by adding or removing values in the range from 0.01 – 0.001 until the genotype prediction was nearly correct with respect to the short-read data. For the genotyping results based on simulation data sets, we compared the predicted and expected number of crossovers. These comparisons suggested that the error rate itself was quite low, below 0.02% for 1x coverage rate.

To avoid over fitting due to the small testing data set, we ran the new model on all samples again and randomly selected ten new samples. For the new ten samples, we again compared the genotype prediction with the support from the short-read data. If the prediction was overall not correct, the emission probabilities were further stepwise changed and a new model was applied to the total sample set again. The procedure was performed until further changing of the emission values resulted in worse genotype predictions. To check the potential over-fitting, the HMMs were applied to a 1,000 new randomly simulated samples with 1x average sequencing coverage. To validate the prediction of our model, we compared the predicted genotypes of AMPRIL data with the previous genotyping result based on 300 SNP markers. For the HMM-2, the probabilities were trained based on the simulation population used for training the HMM-1 but only selecting regions which were heterozygosity. The emission probabilities were adjusted and validated as described above.

Supplementary Table 1. Loss-of-function (LoF) variation, expression (Exp.) and promoter methylation (Met.) of *TAD3* in the AMPRIL parental genomes.

Acc.	TAD3-1 ID	TAD3-1 LoF	TAD3-1 Exp.	TAD3-1 Met.	TAD3-2
An-1	ATAN-5G34920	-	+	-	-
C24	ATC24-5G34790	-	+	-	-
Col-0	AT5G24670	-	+	-	-
Cvi-0	ATCVI-5G35090	-	+	-	-
Eri-1	ATERI-5G35050	-	No RNA-seq data	-	-
Kyo	ATKYO-5G34660	-	No RNA-seq data	+	+
L*er*	ATLER-5G34950	-	+	-	-
Sha	ATSHA-5G34870	-	+	-	-

Supplementary Table 2. The observed and expected frequencies of allele pair combinations of *TIM22-1* and *TIM22-2* in the complete AMPRIL population.

	Observed	***TIM22-1***							
		An-1	C24	Col-0	Cvi-0	Eri-1	Kyo	L*er*	Sha
TIM22-2	An-1	63	41	33	28	52	22	58	26
	C24	13	98	12	17	46	23	36	21
	Col-0	25	18	61	45	27	39	30	22
	Cvi-0	5	13	4	66	12	1	4	18
	Eri-1	38	35	18	23	61	23	18	18
	Kyo	21	27	40	25	21	70	20	35
	L*er*	25	54	18	19	19	17	71	8
	Sha	21	29	16	59	17	58	29	52
	Expected	***TIM22-1***							
		An-1	C24	Col-0	Cvi-0	Eri-1	Kyo	L*er*	Sha
TIM22-2	An-1	69.0	37.9	26.7	29.9	55.0	21.3	64.7	18.6
	C24	12.4	82.3	15.4	27.3	43.3	25.2	37.2	22.9
	Col-0	18.7	25.6	55.7	57.8	27.6	42.7	20.7	18.2
	Cvi-0	10.4	21.0	10.8	32.4	13.3	6.3	10.6	18.2
	Eri-1	34.2	40.5	13.8	28.4	61.3	17.3	17.9	20.6
	Kyo	13.4	26.7	39.0	33.0	16.5	69.1	26.4	34.9
	L*er*	30.3	53.4	19.8	17.7	20.5	17.1	60.0	12.2
	Sha	22.7	27.7	20.7	55.4	17.5	54.1	28.5	54.3

Supplementary Table 3. The observed and expected frequencies of allele pair combinations of *TIM22-1* and *TIM22-2* in the AMPRIL subpopulation EGGE.

	Observed	***TIM22-1***			
		An-1	Col-0	Cvi-0	L*er*
TIM22-2	An-1	26	21	20	11
	Col-0	13	8	2	18
	Cvi-0	1	0	12	1
	L*er*	4	8	12	19
	Expected	***TIM22-1***			
		An-1	Col-0	Cvi-0	L*er*
TIM22-2	An-1	19.5	16.4	20.4	21.7
	Col-0	10.3	8.6	10.7	11.4
	Cvi-0	3.5	2.9	3.7	3.9
	L*er*	10.8	9.0	11.2	12.0

Supplementary Table 4. Loss-of-function (LoF) variation, expression (Exp.) and promoter methylation (Met.) of *TIM22-1* and *TIM22-2* in the AMPRIL parental genomes.

	TIM22-1				***TIM22-2***			
**Acc.**	**Gene ID**	**LoF**	**Exp.**	**Met.**	**Gene ID**	**LoF**	**Exp.**	**Met.**
An-1	ATAN-1G29450	-	+	-	ATAN-3G20030	-	+	-
C24	ATC24-1G29460	-	+	-	ATC24-3G20040	-	+	-
Col-0	AT1G18320	STG	+	-	AT3G10110	-	+	-
Cvi-0	ATCVI-1G29600	-	+	-	ATCVI-3G19910	-	-	+
Eri-1		STG	No RNA-seq data	-	ATERI-3G20040	-	No RNA-seq data	-
Kyo	ATKYO-1G29480	-	No RNA-seq data	-	ATKYO-3G20070	-	No RNA-seq data	-
Ler		STG	+	-	ATLER-3G19980	-	+	-
Sha		STG	+	-	ATSHA-3G25560	-	+	-

STG: stop-codon gain SNP

Supplementary Table 5. Loss-of-function (LoF) variation, expression (Exp.) and promoter methylation (Met.) of *HPA2* and *HPA1* in the AMPRIL parental genomes.

	HPA2				***HPA1***			
**Acc.**	**Gene ID**	**LoF**	**Exp.**	**Met.**	**Gene ID**	**LoF**	**Exp.**	**Met.**
An-1	-	STG	+	-	ATAN-5G20070	-	+	-
C24	ATC24-1G85380	-	-	+	ATC24-5G20050	-	+	-
Col-0	AT1G71920	-	-	+	AT5G10330	-	+	-
Cvi-0	ATCVI-1G86350	-	+	-	-	-	-	-
Eri-1	-	STG	No RNA-seq data	-	ATERI-5G20170	-	No RNA-seq data	-
Kyo	-	STG	No RNA-seq data	-	ATKYO-5G20130	-	No RNA-seq data	-
L*er*	-	STG	+	-	ATLER-5G20130	-	+	-
Sha	-	STG	+	-	ATSHA-5G20120	-	+	-

STG: stop-codon gain SNP

Supplementary Table 6. The observed and expected frequency of allele pair combinations of *HPA2* and *HPA1* in whole AMPRIL population

Observed		***HPA2***							
		An-1	C24	Col-0	Cvi-0	Eri-1	Kyo	Ler	Sha
HPA1	An-1	88	29	16	18	46	20	31	24
	C24	26	68	31	24	32	25	46	21
	Col-0	24	14	69	33	16	40	14	19
	Cvi-0	2	1	14	80	2	11	2	14
	Eri-1	59	44	30	19	78	16	21	24
	Kyo	15	24	65	21	25	83	10	40
	Ler	52	59	33	23	18	29	47	24
	Sha	24	19	19	34	17	40	13	59
Expected		***HPA2***							
		An-1	C24	Col-0	Cvi-0	Eri-1	Kyo	Ler	Sha
HPA1	An-1	79	28	20	30	38	19	35	22
	C24	24	68	35	32	42	19	36	19
	Col-0	22	15	66	37	17	40	14	18
	Cvi-0	12	8	24	33	9	14	8	19
	Eri-1	54	44	32	31	67	21	16	27
	Kyo	19	27	56	21	25	79	13	42
	Ler	56	50	23	34	19	28	50	26
	Sha	25	18	21	35	17	45	12	52

Supplementary Table 7. The observed and expected frequency of allele pair combinations of *HPA2* and *HPA1* in subpopulation ABBA population

Observed		***HPA2***			
		Col-0	Cvi-0	Kyo	Sha
HPA1	Col-0	9	7	6	3
	Cvi-0	9	8	8	5
	Kyo	16	10	9	6
	Sha	5	9	7	9

Expected		***HPA2***			
		Col-0	Cvi-0	Kyo	Sha
HPA1	Col-0	7.2	6.3	7.4	4.2
	Cvi-0	8.6	7.5	8.8	5.1
	Kyo	14.6	12.8	15.0	8.6
	Sha	8.6	7.5	8.8	5.1

Supplementary Table 8. The observed and expected frequency of allele pair combinations of *HPA2* and *HPA1* in subpopulation EFFE population

Observed		***HPA2***			
		Col-0	Cvi-0	Kyo	Sha
HPA1	Col-0	17	4	18	16
	Cvi-0	3	5	3	4
	Kyo	18	11	17	15
	Sha	14	3	16	8
Expected		***HPA2***			
		Col-0	Cvi-0	Kyo	Sha
HPA1	Col-0	16.6	7.4	17.3	13.8
	Cvi-0	4.5	2.0	4.7	3.8
	Kyo	18.4	8.2	19.2	15.3
	Sha	12.4	5.5	12.9	10.3

Supplementary Table 9. Pseudo-heterozygous variants identified in *HPA2* and *HPA1* by RNA-seq and methylation data.

Gene	Variation ID	Chr.	Position	Reference allele	Alternative allele	Functional effect	Number of accessions	Variation could be assigned to *HPA2* or *HPA1*
HPA2	LoF-100	chr1	27,067,175	T	TCAGGGCTCTC	FS	1	*HPA2*
HPA2	LoF-110	chr1	27,067,231	C	T	STG	2	*HPA2*
Both	LoF-120	chr1	27,067,387	ATTTT	ATTT	FS	3	*HPA2*
Both	LoF-120	chr5	3,249,634	TAAAA	TAAA	FS	2	*HPA2*
Both	LoF-130	chr1	27,067,518	C	T	STG	2	Undet.
Both	LoF-130	chr5	3,249,508	G	A	STG	2	Undet.
HPA1	LoF-140	chr5	3,249,497	TC	TCC	FS	1	Undet.
Both	LoF-150	chr1	27,067,590	G	A	spDon	1	Undet.
Both	LoF-150	chr5	3,249,436	C	T	spDon	1	Undet.
HPA1	LoF-160	chr1	27,067,591	C	A	spDon	1	Undet.
Both	LoF-170	chr1	27,067,785	CT	C	FS	210	*HPA2*
Both	LoF-170	chr5	3,249,239	GA	G	FS	212	*HPA2*
Both	LoF-180	chr1	27,067,787	CAA	CA	STG	1	Undet.
Both	LoF-180	chr5	3,249,236	CTT	CT	FS	1	Undet.
Both	LoF-190	chr1	27,067,813	CATACTTGTA	CA	FS	23	Undet.
Both	LoF-190	chr5	3,249,203	CTACAAGTAT	CT	FS	16	Undet.
HPA1	LoF-200	chr5	3,249,064	C	A	STG	1	Undet.
HPA1	LoF-210	chr5	3,249,052	CT	C	FS	1	*HPA2*
Both	LoF-220	chr1	27,067,987	CTTT	CTT	FS	1	*HPA2*
Both	LoF-220	chr5	3,249,035	GAAA	GAA	FS	1	*HPA2*
Both	LoF-230	chr1	27,068,021	TGG	TG	FS	12	*HPA2*
Both	LoF-230	chr5	3,249,002	TCC	TC	FS	13	*HPA2*
Both	LoF-240	chr1	27,068,037	A	T	STG	1	Undet.
Both	LoF-240	chr5	3,248,989	T	A	STG	1	Undet.
Both	LoF-250	chr1	27,068,199	G	T	STG	1	*HPA2*
Both	LoF-250	chr5	3,248,827	C	A	STG	1	*HPA2*
Both	LoF-260	chr1	27,068,344	G	A	spAcc	1	*HPA2*
Both	LoF-260	chr5	3,248,682	C	T	spAcc	1	*HPA2*
Both	LoF-270	chr1	27,068,433	G	T	STG	1	Undet.
Both	LoF-270	chr5	3,248,593	C	A	STG	1	Undet.
Both	LoF-280	chr1	27,068,634	GAA	GAAA	FS	1	*HPA2*
Both	LoF-280	chr5	3,248,392	C	A	STG	1	*HPA2*
Both	LoF-290	chr1	27,068,644	G	A	STG	30	Undet.
Both	LoF-290	chr5	3,248,382	C	T	STG	16	Undet.
Both	LoF-300	chr1	27,068,655	C	T	STG	585	*HPA2*
Both	LoF-300	chr5	3,248,371	G	A	STG	405	*HPA2*
HPA1	LoF-310	chr5	3,248,194	C	A	STG	1	*HPA2*
Both	LoF-320	chr1	27,068,893	TGG	TG	FS	3	Undet.
Both	LoF-320	chr5	3,248,130	ACC	AC	FS	4	Undet.

Both: variation was found in both *HPA2* and *HPA1*; FS: frameshift; STG: premature stop-codon gain; spAcc: splice acceptor site damage; spDon: splice donor site damage; Undet.: undetermined.

Supplementary Table 10. Number of accessions with different combinations of functional (FUN) or non-functional (nFun) *HPA* copies across the 1135 *A. thaliana* accessions of the 1001 Genomes Project. ("Pre" and "Abs" refer to the presence and absence of *HPA3/4* which could only be analyzed for their presence.)

**Allele Combination**	***HPA2***	***HPA1***	***HPA3***	***HPA4***	**Number**
Allele Combination 1	FUN	Abs	Abs	Abs	3
Allele Combination 2	FUN	nFUN	Abs	Abs	1
Allele Combination 3	nFUN	FUN	Abs	Abs	661
Allele Combination 4	FUN	FUN	Abs	Abs	266
Allele Combination 5	FUN	FUN	Pre	Abs	48
Allele Combination 6	nFUN	FUN	Pre	Abs	100
Allele Combination 7	FUN	nFUN	Pre	Abs	1
Allele Combination 8	nFUN	nFUN	Pre	Abs	13
Allele Combination 9	nFUN	FUN	Abs	Pre	6
Allele Combination 10	nFUN	nFUN	Abs	Pre	36

Supplementary Table 11. Number of accessions with different combinations of functional (FUN) or non-functional (nFUN) *TIM22* copies across 1135 *A, thaliana* accessions of the 1001 Genomes Project.

	TIM22-1	***TIM22-2***	**Number**
Allele Combination 1	FUN	FUN	890
Allele Combination 2	FUN	nFUN	4
Allele Combination 3	nFUN	FUN	225
Allele Combination 4	nFUN	nFUN	16

Supplementary Table 12. Loss-of-function (LoF) variation, expression (Exp.) and promoter methylation (Met.) of *FOLT1* and *FOLT2* in the AMPRIL parental genomes.

	FOLT1				***FOLT2***			
**Acc.**	**Gene ID**	**LoF**	**Exp.**	**Met.**	**Gene ID**	**LoF**	**Exp.**	**Met.**
An-1	ATAN-5G89880	-	+	-	ATAN-4G54430	-	+	-
C24	ATC24-5G86520	-	-	+	ATC24-4G57120	-	+	-
Col-0	AT5G66380	-	+	-	-	-	-	-
Cvi-0	ATCVI-5G87930	-	+	-	ATCVI-4G51750	-	+	-
Eri-1	ATERI-5G89000	-	No RNA-seq data	-	ATERI-4G53100	-	No RNA-seq data	-
Kyo	ATKYO-5G89250	-	No RNA-seq data	-	in a contig	-	No RNA-seq data	-
L*er*	ATLER-5G86770	-	+	-	ATLER-4G54710	-	+	-
Sha	ATSHA-5G91420	-	-	+	ATSHA-4G55130	-	+	-

Supplementary Table 13. The number of accessions with different combinations of functional (FUN), non-functional (nFUN) and truncated (trun.) alleles of *FOLT* across 1,135 *A. thaliana* accessions from the 1001 Genomes Project.

**Allele Combination**	***FOLT1***	***FOLT2***	***FOLT2* trun.**	**Number**
Allele Combination 1	FUN	Abs	Abs	195
Allele Combination 2	FUN	FUN	Abs	865
Allele Combination 3	nFUN	FUN	Abs	29
Allele Combination 4	nFUN	FUN	trExon1-2	14
Allele Combination 5	nFUN	FUN	trExon1-6	1
Allele Combination 6	nFUN	FUN	trExon1-2 + trExon1-6	31

Note: trExon1-2 and trExon1-6 indicate that the truncated copy contains the first two or first six exons of *FOLT2*, respectively. “Abs” refers to the absence of intact or truncated *FOLT2*.

Supplementary Table 14. The number of accessions with different combinations of functional alleles of *TAD3* across 1,135 *A. thaliana* accessions from the 1001 Genomes Project

**Functional Allele**	**Number**
TAD3-1	971
TAD3-2	150
TAD3-1, TAD3-2	14

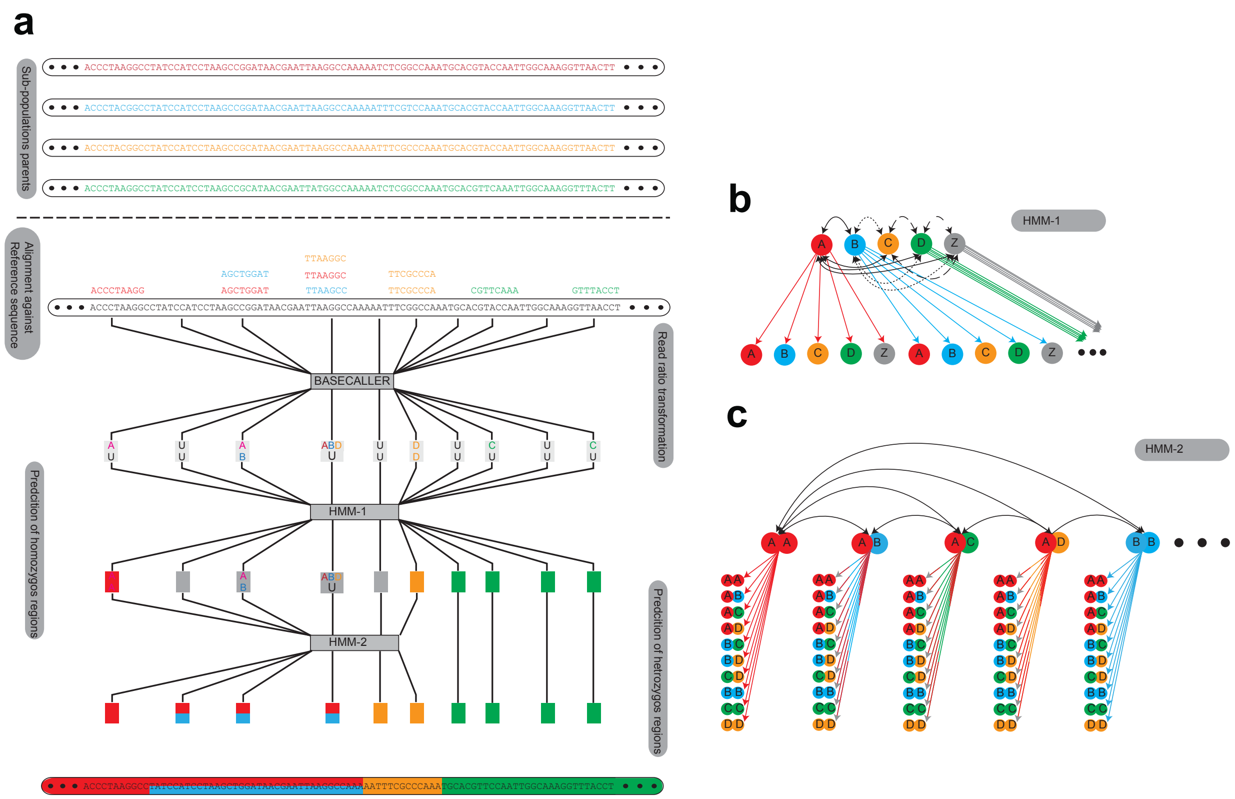

Supplementary Fig. 1 Workflow and HMMs genotyping AMPRIL population. **(a)** Schematic workflow of genotyping. The allele from short read sequencing were translated into genotype calls considering the diploid nature of the genomes. **(b, c)** Hidden State Models of the two HMMs, which were used for genotyping of the AMPRILs. The HMM-Model-1 was used to assign homozygous genotypes, while it did not resolve heterozygous regions (collectively assigned as Z regions). The more complex HMM-Model-2 was then used locally to resolve Z regions into heterozygous states.

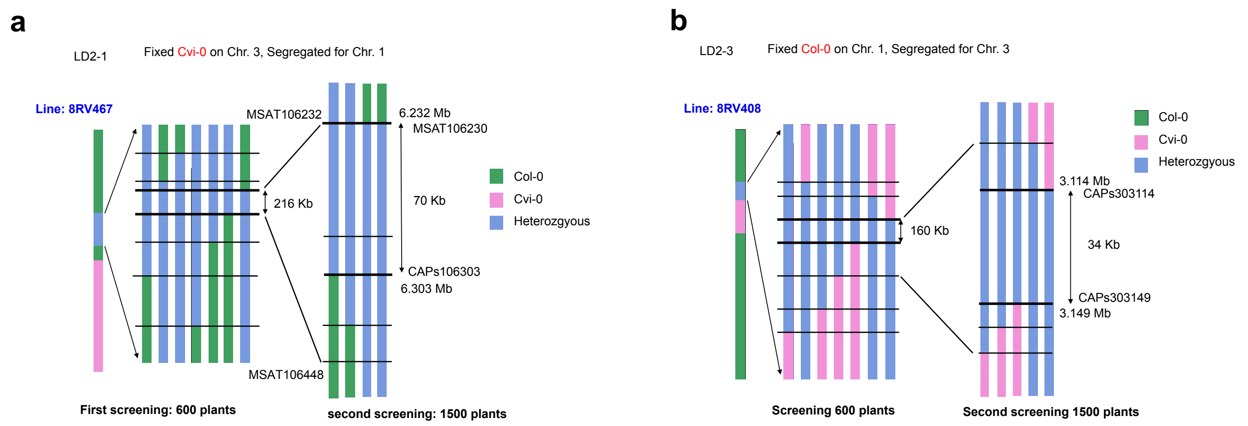

Supplementary Fig. 2 Two rounds of fine-mapping using residual heterozygosity among heterogeneous inbred families from a Cvi-0 x Col-0 RIL population screened genotypically at **(a)** LD2.1 or **(b)** LD2.3 loci.

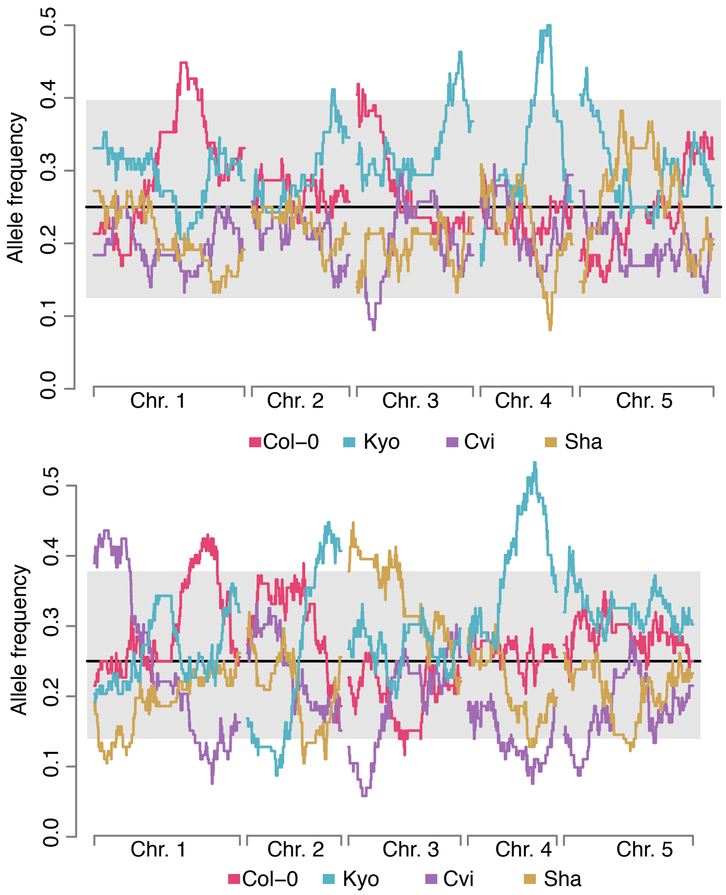

Supplementary Fig. 3 Allele frequency distribution along the chromosome in subpopulation ABBA (above) and EFFE (below)

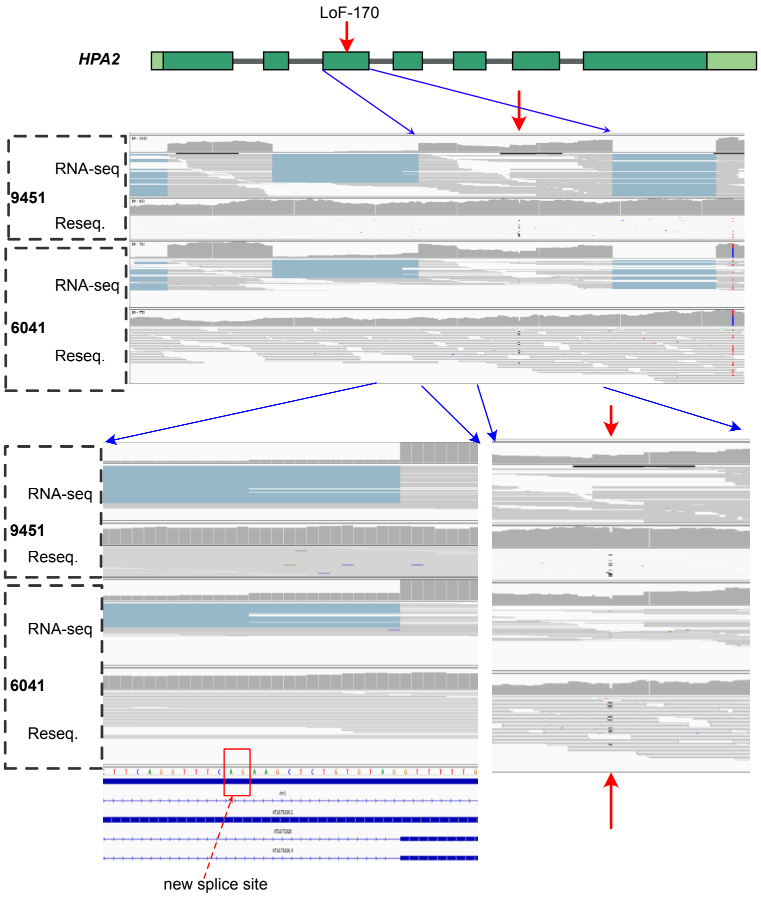

Supplementary Fig. 4 Snapshot of IGV (Thorvaldsdóttir et al. 2013) **of *A. thaliana* accessions with the frameshift mutation (LoF-170) in *HPA2* which was rescued by alternative splicing**. The position of heterozygous variant of LoF-170 is marked with red arrow. In both DNA short read resequencing alignments (Reseq.) of accession Spro 2 (accession ID: 9451) and Lis-3 (accession ID: 6041) the LoF-170 is found. The mutation is however only found in parts of the reads as reads from *HPA1* and *HPA2* are aligned repetitively. However, in RNA-seq read mapping no LoF-170 is found in Spro 2. This suggests that LoF-170 occurred in *HPA2,* because *HPA2* is most likely not expressed due to hypermethylation of its promoter. In contrast, the LoF-170 is still found in RNA-seq reads of accession Lis-3. While this could imply that *HPA2* is expressed, the mutation is linked to an alternative splicing site (indicated by the red dashed arrow) which can complement this frameshift of LoF-170. (Blue regions in RNA-seq read mapping indicate the introns.)

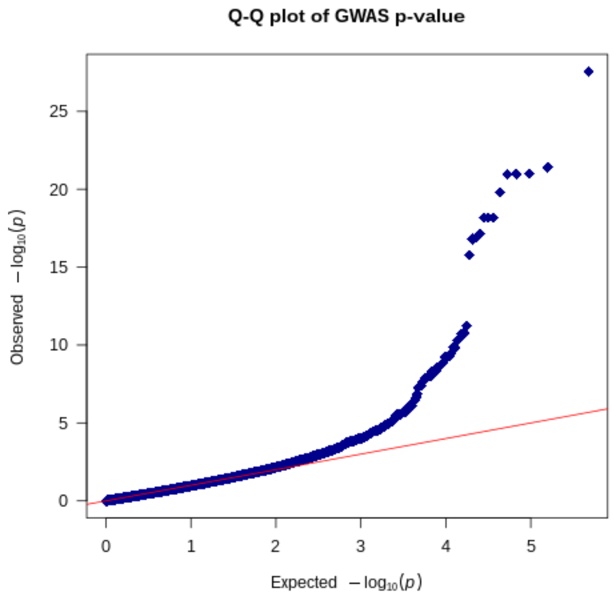

Supplementary Fig. 5. QQ-plot of the GWAS using the absence/presence of functional *HPA* copies as phenotype.

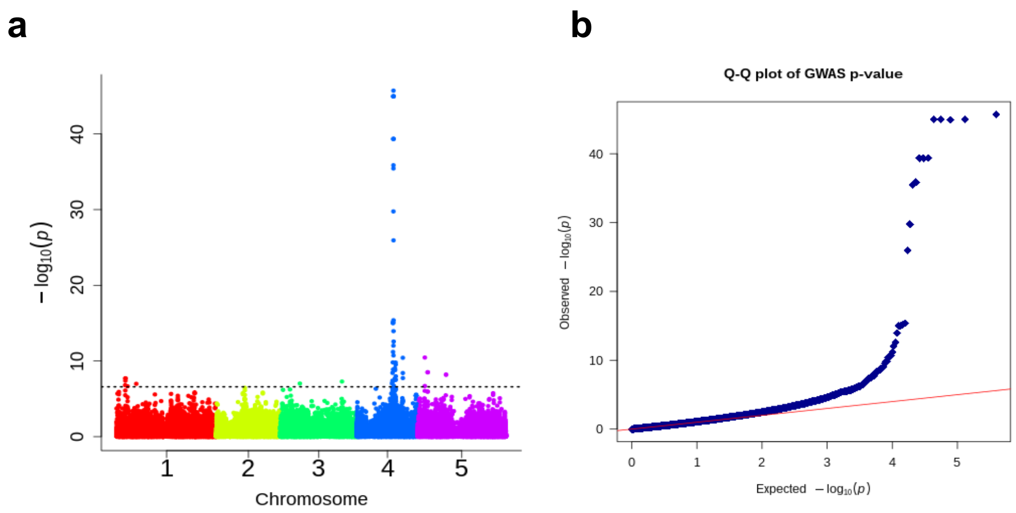

Supplementary Fig. 6. Manhattan plot (a) and QQ-plot (b) of the GWAS using the absence/presence of functional *HPA* copies as phenotype. In contrast to the same approach as shown in Figure 4, samples with *HPA3* were excluded from this analysis.

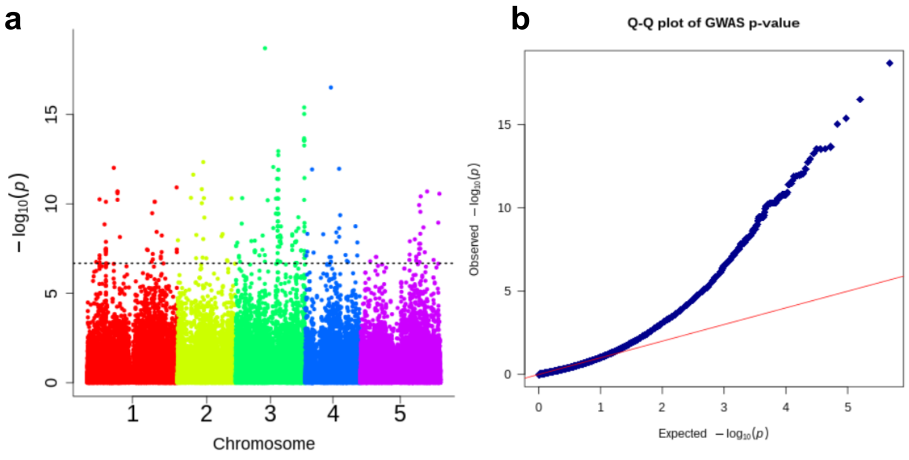

Supplementary Fig. 7 Manhattan plot (a) and QQ-plot (b) of the GWAS using absence/presence of functional *TIM22* copies as phenotype.

a

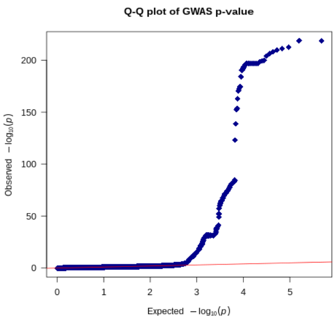

b

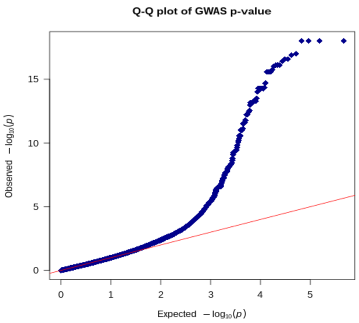

Supplementary Fig. 8 QQ-plot of GWAS for the phenotype of presence or absence of functional *TAD3-1* copy (a) and *FOLT-1* copy (b).

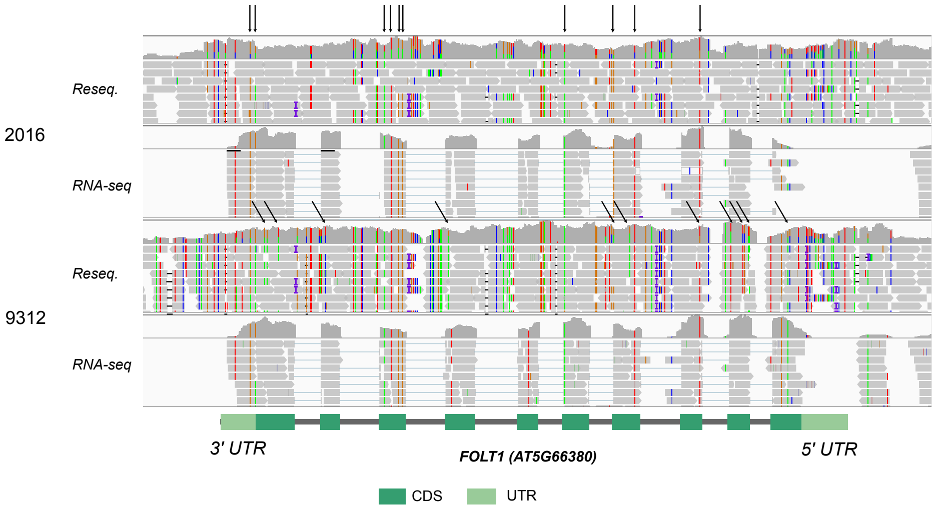

Supplementary Fig. 9 Snapshot of IGV for examples of accessions with expression-silenced *FOLT1* and expression-active *FOLT2*. The heterozygous variants in resequencing reads are marked with arrows, which appear homozygous in the RNA-seq data. Two accessions MNF-Pin-39 (accession ID: 2016) and Ullapool-8 (accession ID: 9312) are used as the examples.

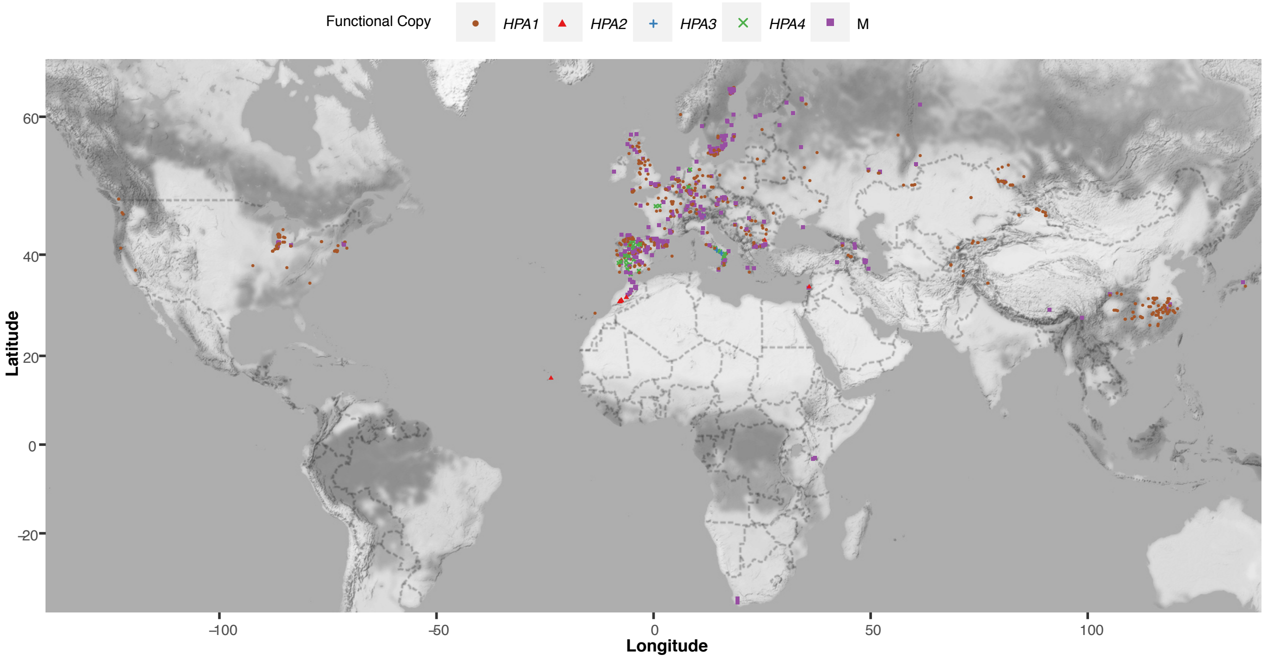

Supplementary Fig. 10 Geographic distribution of functional copies of *HPA* across 1135 *A. thaliana* accessions from 1001 Genomes Project, 75 African accessions and 118 Chinese accessions. M: multiple functional copies, Other accessions only with single functional copy are shown.

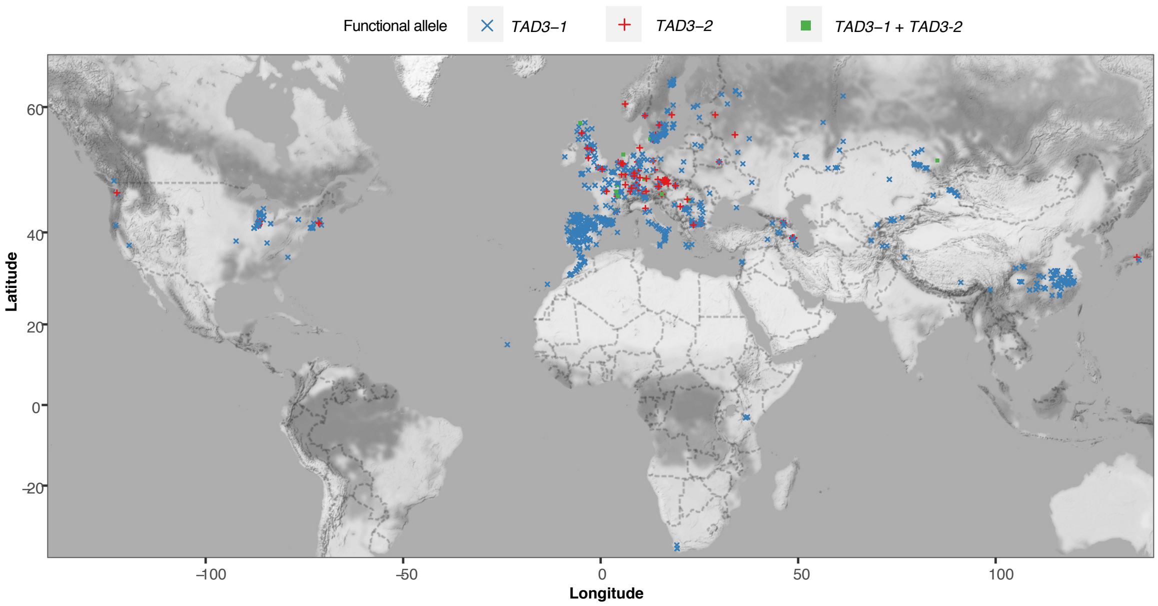

Supplementary Fig. 11 Geographic distribution of functional copies of *TAD3* across 1135 *A. thaliana* accessions from 1001 Genomes Project, 75 African accessions and 118 Chinese accessions.

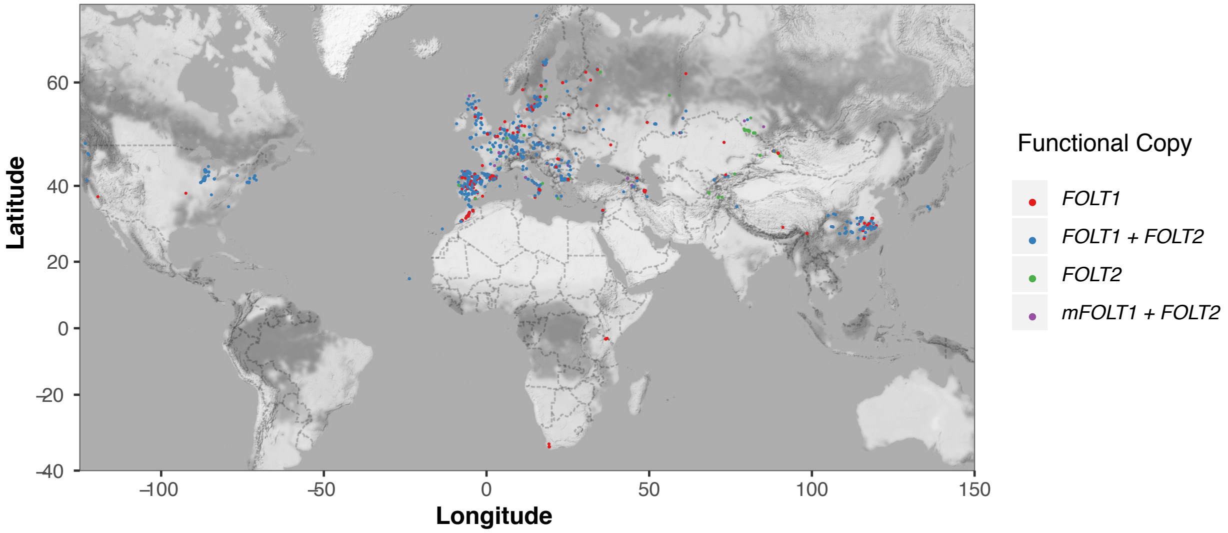

Supplementary Fig. 12 Geographic distribution of functional copies of *FOLT* across 1135 *A. thaliana* accessions from 1001 Genomes Project, 75 African accessions and 118 Chinese accessions. *mFOLT1* + *FOLT2*: accessions with a functional copy *FOLT2* and an unexpressed copy *FOLT1* but without truncated copy of *FOLT*.

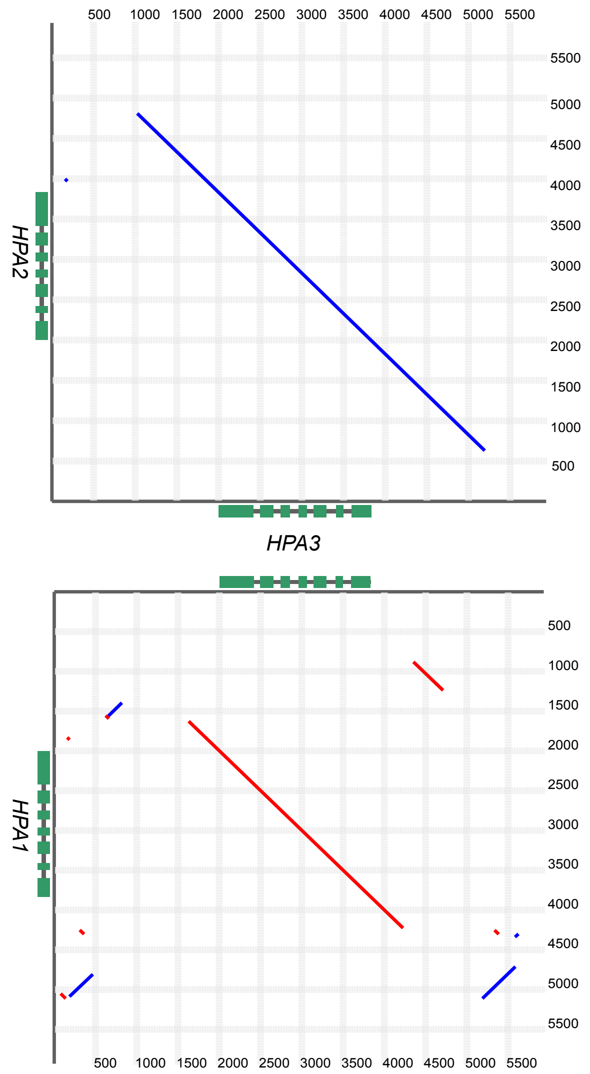

Supplementary Fig. 13 Sequence alignment between *HPA3* and two other *HPA* copies, *HPA1* and *HPA2.*

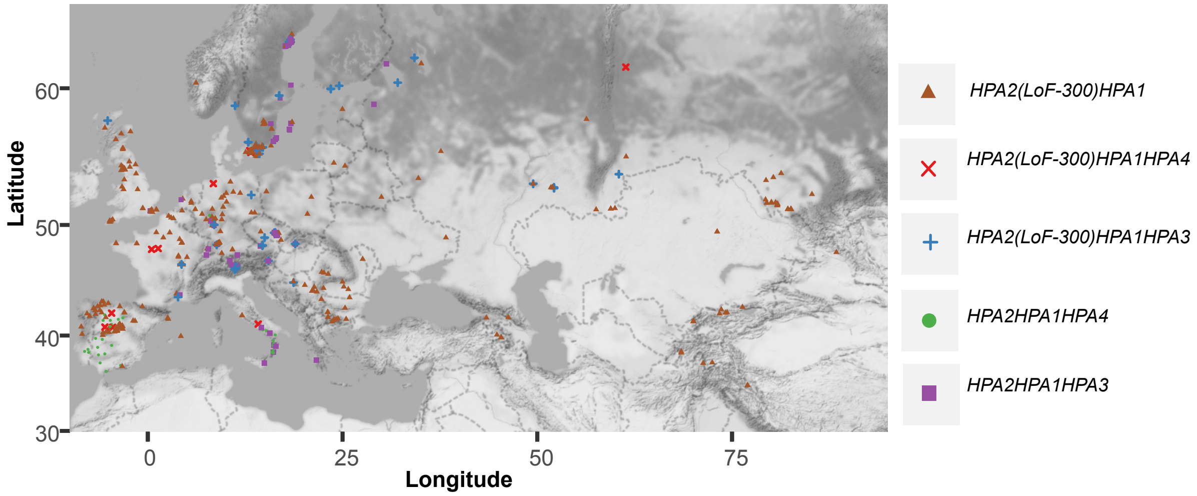

Supplementary Fig. 14 Geographic distribution of Eurasian *A. thaliana* accessions with loss-of-function (LoF) variants in *HPA2* across accessions with the copy of *HPA3* or *HPA4.*

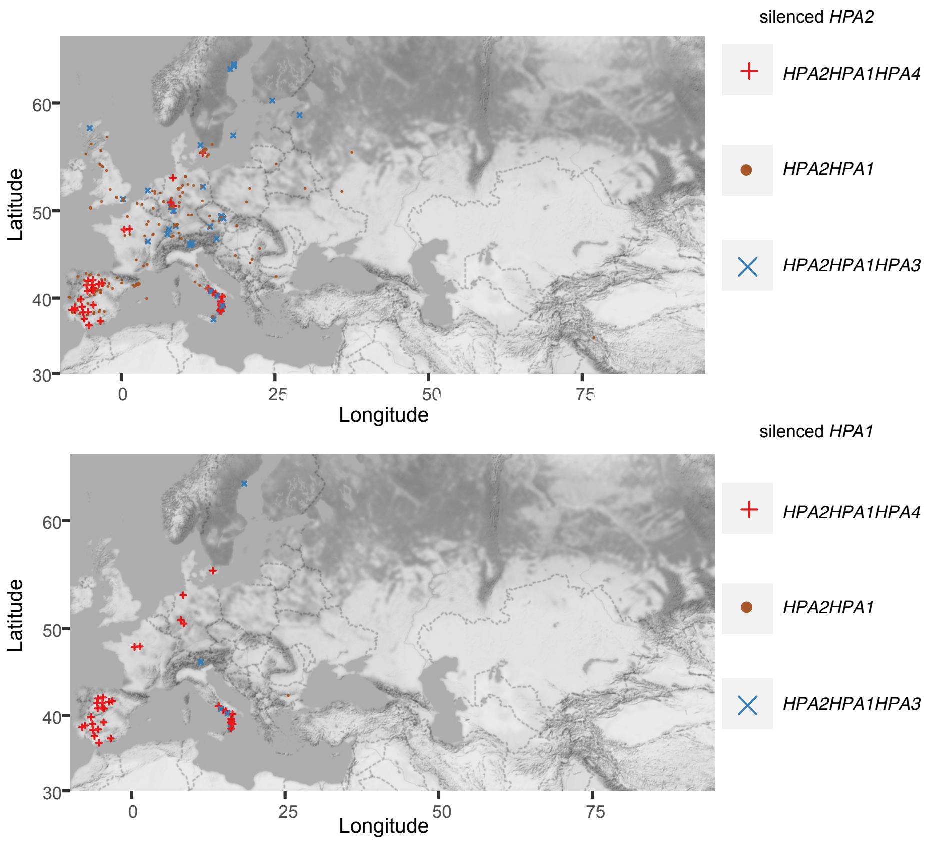

Supplementary Fig. 15 Geographic distribution of *A. thaliana* accessions with different *HPA* copies and methylated promoter in *HPA2* (upper panel) or *HPA1* (lower panel).

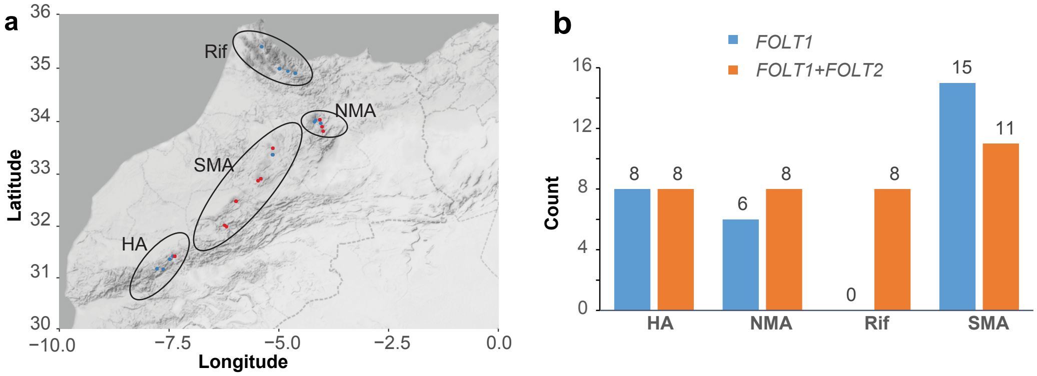

Supplementary Fig. 16 Distribution (a) and number (b) of *FOLT* copies across African accessions.

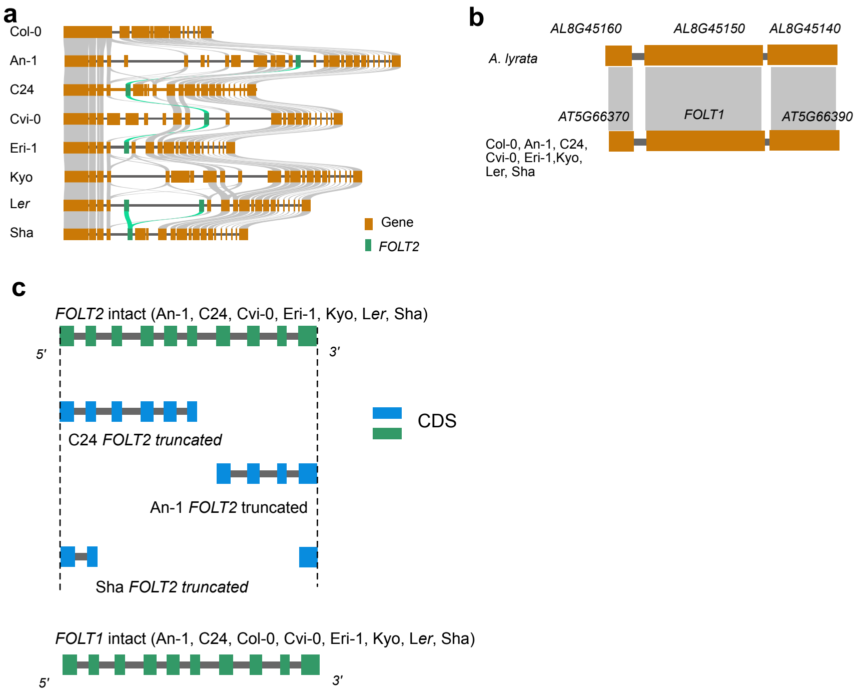

Supplementary Fig. 17 Gene structure of *FOLT* copies in the eight AMPRIL parental genomes as well as in *A. lyrata*. (a) Gene arrangements around the *FOLT2* locus in the AMPRIL eight parental genomes. Gray links indicate orthologous genes. The *FOLT2* gene in Kyo is in a short unanchored contig. **(b)** The *FOLT1* gene in *A. lyrata* and eight *A. thaliana* ecotypes. All of them share syntenic gene arrangement (as indicated by the gray bars). **(c)** The *FOLT2* and *FOLT1* gene structure as observed in the AMPRIL.

Reference

James GV, Patel V, Nordström KJV, Klasen JR, Salomé PA, Weigel D, Schneeberger K. 2013. User guide for mapping-by-sequencing in Arabidopsis. *Genome Biol.* 14.

Rowan BA, Patel V, Weigel D, Schneeberger K. 2015. Rapid and inexpensive whole-genome genotyping-by-sequencing for crossover localization and fine-scale genetic mapping. *G3 Genes, Genomes, Genet.* 5:385–398.

Salomé PA, Bomblies K, Fitz J, Laitinen RAE, Warthmann N, Yant L, Weigel D. 2012. The recombination landscape in Arabidopsis thaliana F2populations. *Heredity (Edinb).* 108:447–455.

Thorvaldsdóttir H, Robinson JT, Mesirov JP. 2013. Integrative Genomics Viewer (IGV): High-performance genomics data visualization and exploration. *Brief. Bioinform.* 14:178–192.
